# The impact of supplementing traditional risk information with polygenic risk score concerning type 2 diabetes and coronary heart disease on health behavior: a randomized controlled trial

**DOI:** 10.1007/s12687-025-00790-7

**Published:** 2025-03-26

**Authors:** Otto Halmesvaara, Marleena Lonna, Helena Kääriäinen, Markus Perola, Kati Kristiansson, Hanna Konttinen

**Affiliations:** 1 Social Psychology, Faculty of Social Sciences, University of Helsinki, Helsinki, Finland; 2 Research Program for Clinical and Molecular Metabolism, Faculty of Medicine, University of Helsinki, Helsinki, Finland; 3 Department of Public Health, Finnish Institute for Health and Welfare, Helsinki, Finland

**Keywords:** Polygenic risk, Type 2 diabetes, Coronary heart disease, Health behavior, Randomized controlled trial

## Abstract

Polygenic risk scores (PRS) for different diseases are expected to become more widely available to the public in the coming decades. In addition to the investigation of the clinical relevance of polygenic risk scores, an assessment of the health behavioral impact is needed. The present study used data from a personalized medicine project that combined genomic and traditional health data to evaluate respondents' risk for common diseases. Specifically, we investigated if supplementing traditional risk estimates of type 2 diabetes and coronary heart disease with PRS influenced respondents' self-reported physical activity, alcohol consumption, fruit/vegetable consumption or prompted the respondents to seek medical treatment/examination. As an exploratory hypothesis, we also tested if there was an interaction between the disease risk level and the experimental/control group for any of the outcomes. A randomized controlled trial was conducted, where the experimental group (*n* = 216 for seeking treatment and 523–459 for other outcomes) received risk estimates based on traditional risk and PRS, and the control group (*n* = 216 and 526–498) based solely on traditional risk factors. On average, approximately 80 days elapsed between the risk disclosure and outcome measurements. We found no significant difference between the groups regarding health behavior (*p*s > .28, *d*s < 0.07) or likelihood of seeking medical treatment/examination (*p* = .86, OR = 1.06). Likewise, no significant interactions were detected (*p*s > .08, *d*s < .11, *OR*s < 1.2). We conclude that we did not find support for either a beneficial or detrimental effect of supplementing traditional risk estimates with PRSs. However, several limitations should be noted when generalizing the results.

**Supplementary Information:**

The online version contains supplementary material available at 10.1007/s12687-025-00790-7.

## Introduction

Type 2 diabetes (T2D) and coronary heart disease (CHD) pose a significant global burden, contributing to widespread disability and premature mortality (Zheng et al. 2018; Sanchis-Gomar et al. 2016). Moreover, the prevalence of diabetes is projected to increase globally in the coming decades, making preventive efforts even more crucial (Ong et al. 2023). While lifestyle factors play a major role and are essential to preventive efforts, genetic predisposition also influences the risk of developing T2D and CHD (Fuschsberger et al. 2016; Roberts and Stewart 2012). Despite the established role of genetics in disease susceptibility, the routine use of polygenic risk scores (PRS) to assess the likelihood of future T2D or CHD is not a standard practice in public healthcare settings. The reasons for this are manifold, ranging from lack of resources to uncertainty of clinical utility (Sud et al 2023; Hingorani et al. 2023). However, advances in genotyping technology and decreased cost have led to expectations that PRSs could become much more common in the healthcare system in the future (Krier et al. 2016; Abul-Husn and Kenny 2019). Moreover, while the utility of PRS in public health care continues to be debated (Sud et al. 2023; Moorthie et al. 2023), direct-to-consumer companies are already offering many PRS-based tests to people, and the industry is expected to grow (Park and Lu 2023). Thus, the PRS will likely be more present in the public sphere, one way or another, in the coming decades.

From a health psychological perspective, and irrespective of the predictive utility of PRS, it is unclear if receiving such risk information benefits health behavior change. For example, early on, Hunter et al. (2008) speculated that disclosing genetic risk scores could more readily motivate participants to follow relevant health recommendations; however, they also warned that low genetic risk could lead to unfounded optimism. A related worry is that higher risk could induce fatalism in recipients (Claassen et al. 2010).

Empirical studies investigating the effects of disclosing genetic information concerning complex diseases are still considerably less common than reports on monogenic mutations. Studies using designs other than randomized controlled trials (RCT) have given preliminary support for the usefulness of genetic scores for health behavior change in the context of complex diseases (Frieser et al. 2018; Widen et al. 2022; see Wallingford et al. 2023 for review). However, most RCTs disclosing PRS have produced nonsignificant or mixed results (e.g., Hollands et al. 2016; Peterson et al. 2018; King et al. 2023). Moreover, considerable heterogeneity exists between the studies. For example, Driver et al. (2022) have claimed that many studies providing PRS information to participants tended to be underpowered, had biased samples, or used uninformative control groups. Likewise, PRSs have been provided for a range of different conditions/diseases with varying target populations and study settings. Some studies have provided extensive interventions with in-depth explanations of the PRS's meaning, while others have been more succinct. Thus, although a preliminary pattern has emerged from studies providing PRS estimates, large-scale RCTs are still a welcomed addition to gain a more complete picture of the PRS's effect in specific settings and for specific conditions. Given that expectations of the usefulness of PRSs are mostly related to the distribution of estimates over a large number of people (as in public healthcare), it seems particularly important for the generalizability of the results that the experimental design corresponds to a situation that can realistically be scaled to mass use.

Consequently, the current study aims to examine how communicating PRS information related to T2D and CHD influences respondents' health behavior in a RCT executed through an internet portal. Using an internet portal with automated delivery is a relatively cost-efficient way to distribute risk estimates to a large group of people and is scalable to high volumes. T2D and CHD were selected as suitable conditions for the study since both are common diseases in Finland, and risk mitigation is possible through lifestyle changes (Abouzeid et al. 2015; Salomaa et al. 2016). Furthermore, genome-wide polygenic scores have been published for both conditions (Khera et al. 2018).

Health behaviors selected for the study were physical activity, alcohol consumption, and diet (related to vegetable and fruit consumption), which are all known protective or risk-increasing factors for the development of T2D/CHD (Zheng et al. 2018; Vaduganathan et al. 2022). So far, only a handful of RCTs have been conducted in which the effect of receiving T2D or CHD PRS estimates has been investigated in relation to physical activity, alcohol consumption, and/or vegetable and fruit consumption (Voils et al. 2015; Godino et al. 2016; Kullo et al. 2016; Knowles et al. 2017; Viigimaa et al. 2022). In general, the studies have not been able to detect beneficial or detrimental effects concerning health behavior compared to active control groups.

In addition to typical health behavior measures, we also wanted to investigate whether the polygenic risk information might prompt the respondents to seek medical treatment or examination for the conditions more easily — a question that has not received much attention thus far. From a public health perspective, seeking medical treatment more readily could be beneficial if treatment is warranted. However, if there is no real need for the visit, it could also lead to an unnecessary burden for the public healthcare system, which is a concern that has been expressed if PRS would become widely available for patients (Sud et al. 2023).

Our primary research question is:

1. Does receiving T2D- and CHD-related risk estimates based on a combination of genome-wide polygenic and traditional risk factors influence physical activity, alcohol consumption, vegetable and fruit consumption, or likelihood of seeking medical treatment/examination differently than receiving estimates based solely on traditional risk factors?

On a more exploratory note, we also test:

2. Does the effect of receiving genome-wide polygenic and traditional risk estimates depend on the level of the overall T2D risk or the CHD risk (i.e., if there is an interaction between the T2D or CHD risk level and the experimental/control group)?

## Methods

### Overview

A RCT was conducted in which the experimental group received T2D/CHD risk estimates based on PRS and traditional risk factors, and the control group received estimates based on traditional risk factors only. Four outcome measures (from two different surveys) were tested, of which three (PA, alcohol consumption, vegetable/fruit consumption; n = 1049–957) were measured, on average, 78 days after the disclosure of the risk estimates, and one (whether the participant sought medical treatment/examination; n = 421) was measured, on average, 81 days after the disclosure. PA, alcohol consumption, and fruit and vegetable intake were selected as appropriate outcome measures, as they are established protective factors against developing T2D/CHD (with reduced alcohol consumption being protective in the case of alcohol). An internet portal was used to disclose the risk estimates and collect the respondents' self-reported data on the outcomes. Participants received their risk scores presented as a numerical value, accompanied by a brief written explanation of the score, and as a visual graph indicating whether the risk was low, elevated, high, or very high. Additionally, all participants were provided with a health report emphasizing the importance of physical activity, a healthy diet, maintaining a normal weight, and avoiding smoking to reduce the risk of T2D/CHD. The data was analyzed using different regression models. Per-protocol (PP) type analyses were selected as the preferred analysis to be reported. Sensitivity analyses included intention-to-treat (ITT) type analyses and analyses with multiple imputed (MI) data.

### Participants

The present study used data from a sub-section of the P5 study.^1^P5 was a personalized medicine project led by the Finnish Institute for Health and Welfare (THL). The project combined genomic and traditional health data to evaluate participants’ risk for T2D, CHD, and venous thromboembolism (VTE). It then utilized an internet portal to communicate the estimated future disease risks (see Marjonen et al. 2021). Initially, participants were recruited from a population based FinHealth 2017 Survey (Borodulin and Sääksjärvi 2019) to participate in the P5 study. Respondents who had consented to THL biobank, given a blood sample, and had a working internet connection were accepted to participate (out of 6189 respondents, 3449 consented, and 3177 had sufficient data to be included in the randomization).

The main part of the P5 study consisted of four surveys: Survey 1 (S1; which had to be returned before respondents could see their risk estimates), Survey 2 (S2), which became available in the internet portal immediately after S1 was returned, Survey 3 (S3), which became available approximately three months after S1 was released, and Survey 4 (S4), which was available immediately after S3 was returned (see Supplementary File 1 for more details). After S3, respondents in the control group gained access to their genetic risk scores, thus ending the proper RCT part of the study.

The current study used data from two different surveys collected during the P5 project. Three of the outcomes, namely PA (MET minutes), alcohol consumption, and vegetable and fruit consumption, were derived from S3 (n = 1200), and one outcome, whether the respondent sought medical treatment/examination due to the risk estimates received, was obtained from S4 (*n* = 471).^2^

### Study design

Due to issues with the internet portal and the timing used to collect the data, we did not have a valid baseline measure for most of the participants.^3^ Thus, no baseline was used, and a posttest-only control group design was utilized for all analyses, which is less efficient but a valid design to estimate the average treatment effect (O'Connell et al. 2017).

### Intervention

Participants were randomized into experimental and control groups. Both groups received future disease risk estimates based on respondents’ risk of developing T2D/CHD during the next ten years. T2D risk was categorized into four levels (below 7.5% risk as low, 7.5–10% as elevated, 10–20% as high, and more than 20% as very high) and CHD risk in 3 levels (below 7.5% as low, 7.5–10% as elevated, and more than 10% as high). Along with the T2D and CHD estimates, the respondents also received information concerning their non-genetic risk for developing venous thromboembolism (VTE)^4^ and information on selected single clinical variants (SCV) related to CHD and VTE (see Marjonen et al. 2021 for details).

The experimental group received disease risk estimates based on traditional risk factors and PRS, and the control arm received estimates based solely on the traditional risk factors. Traditional risk factors included BMI, total cholesterol, high-density lipoprotein, systolic blood pressure, blood pressure-lowering medication, lipid-lowering medication, self-reported family history, and smoking status. We used polygenic scores, previously published in the UK Biobank population, containing close to 7 million genomic variants for T2D and 6.6 million variants for CHD (Khera et al. 2018; see Marjonen et al. 2021 for details concerning the calculation).

The risk scores were presented as a number, accompanied by a short-written description of the risk score, and as a graph where the participant’s risk was contrasted to the above-mentioned risk levels for the relevant disease (see Marjonen et al. 2021 for examples). In addition to the 10-year combined disease risk, the experimental group was provided with a graph showing their PRSs separately. Also, a health report (approximately 500–600 words) was given to all participants, explaining the results more thoroughly and providing instructions on how participants could influence their disease risk with lifestyle changes. The health report was tailored slightly based on risk and age groups, but generally, respondents were encouraged to adopt lifestyle changes to reduce their T2D/CHD risk. For example, if participants had a low risk for T2D, the report included the statement: “Based on our estimate, your risk of developing type 2 diabetes within the next ten years is less than 5%. In your age group, this is a fairly low risk compared to the average population risk. […] a healthy lifestyle can further reduce your risk of developing a disease or postpone the onset of a disease by several years. It is particularly important to engage in physical activity, eat healthily and aim at maintaining a normal weight. Even a minor weight loss will reduce the risk. It is also important not to smoke. Good instructions are available at the Finnish Diabetes Association website at […]”. Moreover, for those with elevated/higher risk, the following guidance was included in the report: “You can show/We recommend that you show this message to a physician or public health nurse at a health centre or in occupational health care, for instance, to receive guidance and support for implementing lifestyle changes.” (See Supplementary File 2 for complete example). Finally, an interactive calculator was introduced, where respondents could test how changing their physical parameters (e.g., age) and lifestyle factors (e.g., smoking) would change their risk score.

### Outcomes

#### Physical activity (MET minutes)

MET minutes refer to the metabolic equivalent of task minutes, where 1 MET minute approximates the energy expenditure of sitting quietly for 1 min (Haskell et al. 2007). MET minutes allow a rough translation of different intensity grades of PA into a single scale and are widely used when self-reported PA is measured.

Respondents were asked to estimate the average number of hours and minutes of low-intensity, moderate-intensity, and high-intensity PA they have done per week in the last three weeks. Before calculating the MET score, we excluded, at face value, respondents who claimed they had 12 h or more PA on each day during the last three weeks (n = 2). MET coefficients were obtained from approximations suggested by Haskell et al. (2007), where common low-intensity PA activities had a MET coefficient of 1.5–3, moderate-intensity activities had a MET coefficient of 3–6, and high-intensity activities had a MET coefficient of 6–11.5. We used the midpoint of the suggested range for low-intensity and moderate-intensity PA. However, given the higher range in suggested MET values for high-intensity activities, we were more conservative and used the lower quartile as an approximation for the met coefficient. Thus, the selected approximations for MET values were 2.25 for low-intensity activities, 4.5 for moderate, and 7.375 for high-intensity PA. For example, if the respondent reported 420 min of low-intensity, 210 min of moderate-intensity, and 60 min of high-intensity PA on average per week, the associated MET minutes were 2.25 × 420 + 4.5 × 210 + 60 × 7.375 = 2332.5 per week.

#### Alcohol consumption

Average alcohol consumption was assessed using two different questions. Respondents were asked how often they, on average, consumed alcohol (ranging from abstinence to 4 times or more a week) and the average amount of alcohol consumed per drinking session (from 1–2 servings to more than 10 servings per session). Servings were defined as 12 g of pure alcohol (examples were given to illustrate how much one portion is). Then, to obtain a continuous measure of average portions per week, each question's midpoint (converted to weekly consumption) was taken, and the numbers were multiplied by each other. For example, 2–4 drinking times per month with 5–6 servings per occasion translated into 0.75 × 5.5 = 4.125 portions per week (i.e., midpoint from 2–4 is 3, and 3 times a month converted into weekly consumption is 3 / 4 = 0.75, which is then multiplied with the midpoint of 5–6 servings).

#### Vegetable and fruit consumption

Vegetable and fruit (including berries) consumption was measured using two questions. 1) "How often have you eaten fruit or berries within the past 7 days?" and 2) "How often have you eaten vegetables (not including potatoes) within the previous 7 days as such, grated or in a fresh salad?". Both had similar response scales: "1 = Not at all, 2 = 1 to 2, 3 = 3 to 5 days, 4 = on 6 to 7 days, 5 = Several times a day". As both vegetable and fruit consumption are similarly related to T2D and CHD risk (Wang et al. 2016; Zurbau et al. 2020) and showed a reasonable correlation with each other (r = 0.44; alpha = 0.61), we created a simple composite score that used the mean of the two variables.

#### Seeking medical treatment/examination

Whether the participant had sought medical treatment/examination was measured with one question: "Have you sought a medical examination or treatment (with a physician, a public health nurse or a nurse) based on the feedback you received from the P5 study? (1 = No, I have not, 2 = I might do that, 3 = Yes, I have)". Since we were interested only in actual behavior and due to the previously mentioned issues with the S4 measure point, we classified participants who answered that they might seek medical treatment as "No" answers (n = 13).

All the outcome measures, excluding if the participant sought treatment/examination, were adapted from the FinHealth 2017 survey (Borodulin and Sääksjärvi 2019). The exact wordings, calculations, and distribution plots for the measures and the subitems can be found in the Supplementary File 3. Raw descriptive statistics per experimental/control group are displayed in Tables 1 and 2.

**Table 1:** Descriptive statistics for the experimental (G + T) and the control (T) group

Outcome	*n*		mean		sd		median		range	
	G + T	T	G + T	**T**	G + T	T	G + T	T	G + T	T
**MET minutes**	474	500	2012	2038	1811	2093	1560	1560	10635	16620
**Alcohol consumption**	459	498	2.88	2.74	4.2	3.97	1.25	1.25	32	25
**Vegetable and fruit consumption**	523	526	3.51	3.51	0.84	0.83	3.5	3.5	4	4
**Seeking medical treatment/examination**	216	205	**G + T Yes/No**: 19/197; **T Yes/No**: 17/188							

"G+T" (Genetic+Traditional) refers to the experimental group, and "T" (Traditional) to the control group. Since "Seeking medical treatment/examination" is a binary variable, proportions in absolute numbers are presented instead of typical descriptive statistics. MET minutes are rounded to zero decimal places, and all other variables to 2 decimal places

**Table 2:** The sociodemographic characteristics of respondents at three different time points: after randomization, in Survey 3, and in Survey 4

Variable	Category	After randomization	Survey 3	Survey 4
**N**	Total (G + T/T)	3177 (1587/1590)	1200 (609/591)	471 (245/226)
**Gender (%)**	Woman	56 (55.5/56.5)	56.1 (54/58.2)	58.8 (56.3/61.5)
**Age Group (%)**	< 30	6.7 (7/6.5)	7.1 (8.4/5.8)	5.1 (6.1/4)
	30–39	13.4 (12.4/14.5)	16.4 (17.2/15.6)	16.1 (17.6/14.6)
	40–49	15.3 (15.6/15)	18.5 (18.4/18.6)	17.8 (18.4/17.3)
	50–59	21.7 (22.4/21.1)	25.8 (25.5/26.1)	27.4 (25.3/29.6)
	60–69	25.8 (26/25.5)	24.3 (23.3/25.4)	25.9 (25.3/26.5)
	70–79	14.2 (14.4/14)	7.2 (7.2/7.3)	7.4 (7.3/7.5)
	80 >	2.9 (2.3/3.5)	0.7 (0/1.4)	0.2 (0/0.4)
**Educational level (%)**	Comprehensive	15.2 (15.3/15)	9.3 (9/9.5)	7.4 (6.5/8.4)
	Intermediate	32.8 (33.7/32)	30.3 (32.1/28.4)	29.5 (33.5/25.2)
	Higher	52 (50.9/53)	60.5 (58.9/62.1)	63.1 (60/66.4)
**Occupational status (%)**	Employed	52.3 (53.6/51)	62.1 (64.5/59.7)	59.9 (63.3/56.2)
	Unemployed	4.8 (4.7/5)	4.9 (4.9/4.9)	5.9 (5.7/6.2)
	Pensioner	36.3 (35.8/36.7)	25.4 (24.5/26.4)	27.2 (26.1/28.3)
	Student	3.3 (3/3.5)	3.8 (3.6/4.1)	3.2 (2.4/4)
	Other	3.3 (2.8/3.8)	3.7 (2.5/4.9)	3.8 (2.4/5.3)
**Annual income (%)**	25,000 € or less	19.1 (18.7/19.4)	13.8 (13.9/13.6)	13.7 (14/13.4)
	25,001—45,000	26.6 (27.4/25.7)	22.9 (22.1/23.8)	19.5 (19.8/19.2)
	45,001—60,000	18.8 (18.2/19.4)	20 (20.1/20)	22.9 (21.4/24.6)
	60,001—80,000	17.4 (16.8/18)	20.3 (19.4/21.2)	18 (18.5/17.4)
	Over 80,000 €	18.2 (18.9/17.4)	23 (24.5/21.4)	25.9 (26.3/25.4)
**Marital status (%)**	Has a partner	73.6 (72.5/74.7)	75.8 (75.7/75.9)	75.1 (74.6/75.6)
	Divorcee or widow	15 (15.4/14.6)	12.3 (11.7/12.9)	11.9 (12.3/11.6)
	Single	11.4 (12.1/10.7)	11.9 (12.7/11.2)	13 (13.1/12.9)
**Has no children (%)**		21.5 (22.3/20.6)	23.4 (24.6/22.1)	25.9 (25.8/25.9)
**T2D risk (%)**	Low	69.6 (70.4/68.7)	73.1 (74.5/71.6)	73.2 (73.9/72.6)
	Elevated	7.8 (7.2/8.4)	7.7 (7.6/7.8)	7.4 (6.1/8.8)
	High	14.1 (13.7/14.4)	12.2 (11.7/12.9)	11.7 (13.1/10.2)
	Very high	8.5 (8.6/8.4)	7 (6.2/7.8)	7.6 (6.9/8.4)
**CHD risk (%)**	Low	70.9 (70.7/71)	78.8 (78.8/78.8)	79.8 (78/81.9)
	Elevated	7.5 (7.4/7.6)	5.9 (5.6/6.3)	6.6 (6.5/6.6)
	High	21.6 (21.9/21.4)	15.2 (15.6/14.9)	13.6 (15.5/11.5)

The first number is the overall percentage in the sample associated with a particular sociodemographic factor, and the figures in brackets are the percentages for the same factor in the experimental ("G+T"; Genetic+Traditional) and control ("T"; Traditional) groups. For example, in the column "After randomization," the row "Gender (%): Women" states that after randomization, the percentage of women was 56 in the total sample, 55.5 in the experimental group, and 56.5 in the control group

### Statistical analysis

As the main goal was to examine whether adding the PRS would induce any benefits over providing just the traditional risk factors to participants, our primary analysis focused on the overall difference between the experimental and control group for each outcome. Suitable regression models were calculated for each outcome, and marginal means were estimated from the models and compared using z-tests (excluding “Vegetable and fruit consumption” where t-test was used).

As MET minutes and alcohol consumption displayed notable skewness with a long tail, we decided to use robust linear regression with MM-estimator, which has a high breakdown point and is thus more resistant to regression outliers (Wilcox 2022). We also accompanied the analysis with an estimate of the median difference using a trimmed Harrell-Davis quantile estimator with percentile bootstrap (Wilcox 2022; Akinshin 2022). Vegetable and fruit consumption was estimated using ordinary least squares (OLS) regression. Whether the respondent sought treatment/examination was estimated with a generalized linear model (GLM) using binomial distribution and logit link. Due to the relatively small sample size and rare outcome (32 out of 375 had sought medical examination/treatment), we used a Firth-type penalized maximum likelihood estimator in the GLM model (Rainey and McCaskey 2021).

As an exploratory analysis, we also investigated if the 10-year T2D or CHD risk level interacted with the experimental/control group. Both the CHD and T2D risk were recategorized into two classes, with either "Low" or "Higher" risk. That is, the risk for T2D collapsed from four levels into two (below 7.5% / more than 7.5%), and likewise, CHD risk collapsed from three levels into two (below 7.5% / more than 7.5%). Then, two regression models (using the above-mentioned specifications) were calculated for each outcome, one with experimental/control group interaction by T2D risk level and one with interaction by CHD risk level. Heteroscedasticity-consistent standard errors were used in all linear regression models (HC3 for OLS and Croux et al. 2003 standard errors for the robust models).

Finally, due to the many issues with the data (i.e., lack of suitable baseline, many respondents having an inappropriate time interval between surveys, and relatively noise-prone outcome measures), PP analysis was chosen as the primary approach to see if the treatment effect would exist in "ideal" conditions. However, as PP analysis can introduce additional bias, we also estimated the effects with a modified ITT framework (ITT with complete cases). Moreover, given that complete case analysis assumes that respondents are missing completely at random, we also estimated the PP and ITT effect with MI data.

Note that the terms ITT and PP are used here in a very loose sense. ITT simply refers to models with no deletions, and PP refers to models where deletions were made to ensure that the outcome measures measured the effect of receiving the risk estimates.

All analyses were conducted with R v. 4.3.1 (R Core Team 2023) and RStudio v. 1.9.494 (Posit team 2023). See Supplementary File 4 for detailed list concerning the packages used.

### Sensitivity power analysis

As the number of respondents was based on the number of available volunteers from the FinHealth survey, prospective power calculation was not sensible. Thus, we performed a sensitivity power analysis to get an estimate of how small the minimum detectable effects with 80% power would be for a two-sided test with 0.05 alpha. For the primary analysis, we used the G*power software (Faul et al. 2007) to estimate the minimum detectable effect when using a t-test or a large sample z-test. Cohen's d for the treatment/control difference ranged from 0.17 to 0.18 for the linear regression models, and the OR for the logistic model was estimated to be 2.3. For the exploratory analysis (i.e., the interaction effects), we used the R package InteractionPoweR (Baranger et al. 2023), which utilizes correlation-based simulations to estimate the power of interaction effects. The effect size was estimated as a correlation (Pearson's correlation coefficient r) between the interaction term and the outcome variable. Here, the linear models' effect sizes ranged from r = 0.14 to 0.15, and the logistic model's effect size was r = 0.27 for both risk factors. See the Supplementary File 5 for a more detailed explanation.

The estimates from the sensitivity power analysis indicated that the study should not have been underpowered to detect even relatively minor differences between the experiment and the control group. However, we also encourage the readers to inspect the 95% confidence intervals around the effect size estimates to get a better sense of the range of effects most compatible with the data (Hoenig and Heisey 2001; Dziak et al. 2020).

## Results

### Response rates and intervals

Since we could not precisely control when the respondents returned each survey, we calculated the response interval between S1 and S3/4 to estimate the average follow-up period per experimental/control group. The response interval (in days) between returning the first and third/fourth surveys (S1-3/S1-4) was very similar between the experimental and control group (the median and the mode for S1-3 and S1-4 were 86 days for both groups, and only slight differences were observed between the means, 76 vs 80 for S1-3 and 80 vs 83 for S1-4; see Supplementary File 1 for complete results). Thus, on average, it took 78 days after seeing the results to return S3 and 81 days to return S4. It is also noteworthy that the response interval did not notably correlate with any outcome measures, indicating that the average response to the risk estimates stayed the same irrespective of how much time elapsed after seeing the risk information (τ < 0.02 and r < 0.04, ps > 0.20, LOESS-regression likewise indicated near flat curve; see Supplementary File 1 for details).

**Fig. 1:**
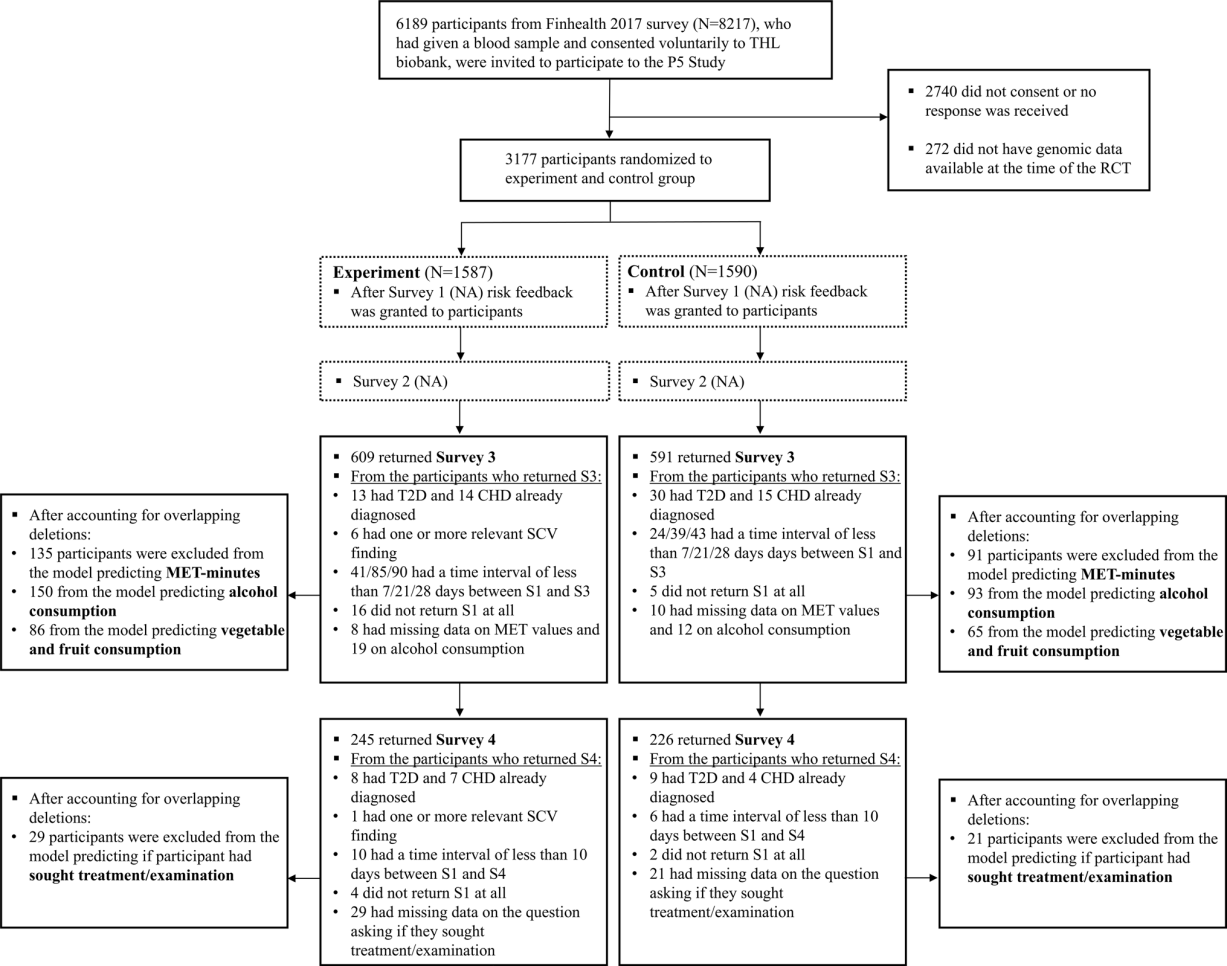
Flow-chart concerning the sample used: Response rates and exclusions

Figure 1 provides a general breakdown of response rates for each survey used in the primary analysis and the deletions concerning each model per experimental/control group. Participants were excluded from the analysis if they had inappropriate time interval between surveys (S1-3 < 7 d for fruit and vegetable intake, S1-3 < 21 d for PA and alcohol consumption, and S1-4 < 10 d for seeking treatment), were carriers of SCV relating to CHD/VTE, or had T2D/CHD already diagnosed (See Supplementary File 1 for more details). Table 1 presents respondents’ sociodemographic characteristics after randomization and in the two surveys used (i.e., S3 and S4). The attrition after randomization was high (approximately 62% for S3 and 87% for S4), but differential attrition between the experimental and control groups remained low (see Supplementary File 1 for a more detailed description).

### Effect of the experimental/control group on the outcome measures

#### Physical activity (MET minutes)

The estimated mean for the experimental group was 1666 (SE = 67) and 1620 (SE = 65) for the control group. When the estimated means concerning MET minutes were compared, no significant difference between the groups was observed (MD = 46, SE = 93, *p* = 0.62, d = 0.03). Likewise, no significant difference in the estimated medians was noted (MD = −10, *p* = 0.94).

#### Alcohol consumption

The estimated average portions for the experimental group were 1.46 (SE = 0.08) and 1.34 (SE = 0.08) for the control group. No significant difference between the groups emerged regarding estimated means (MD = 0.12, SE = 0.11, p = 0.28, d = 0.07) or medians (MD = 0.001, *p* = 0.36) of average alcohol consumption.

#### Fruit and vegetable consumption

The estimated mean concerning the composite score was 3.51 (SE = 0.04) for the experimental group and 3.51 (SE = 0.04) for the control group. No significant difference between the estimated group means was noted for vegetable and fruit consumption (MD = −0.001, SE = 0.05, *p* = 0.99, d =  < 0.001).

#### Seeking medical treatment/examination

The probability of seeking treatment/examination was 0.090 (SE = 0.019) for the experimental group and 0.085 (SE = 0.019) for the control group. There was no significant difference between the groups (OR = 1.06, *p* = 0.86).

**Fig. 2:**
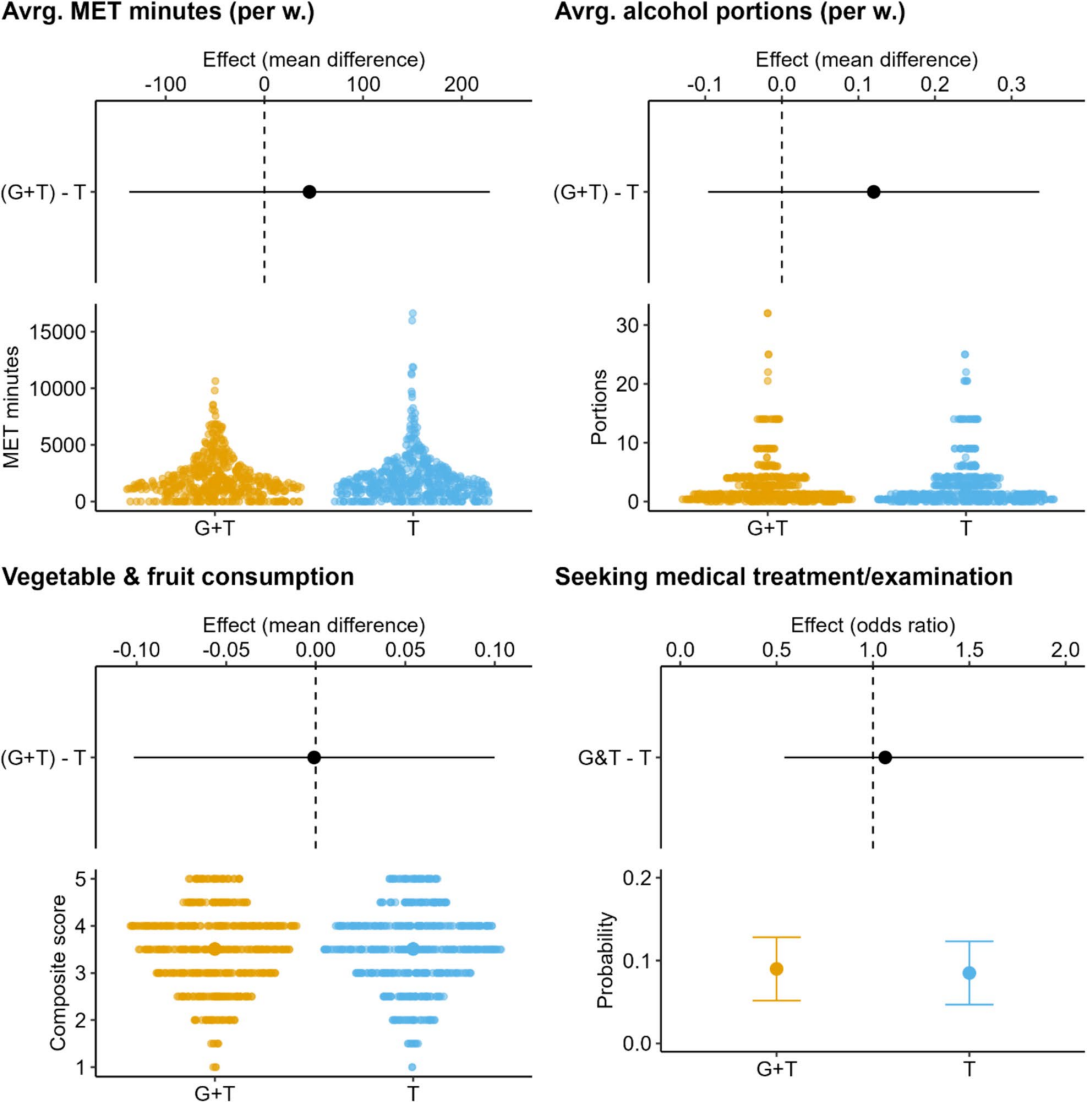
Estimated effect (mean difference or odds ratio) for the experimental/control group. Note. The effect refers to the unstandardized mean difference between the experimental (“G+T”; Genetic+Traditional) and the control (“T”; Traditional) group (excluding the seeking medical treatment/examination variable, where the effect is the odds ratio). For example, the effect for MET minutes was 30, indicating that the experimental group's estimated mean was 30 MET minutes higher than the control group's. The interval around the point estimate is a 95%confidence interval

See Fig. 2 for a visual representation of the results and Table 3 for the estimated means and 95% confidence intervals for all statistics.

**Table 3:** Estimated marginal means and effect sizes with 95% confidence intervals for the mean/median differences between the experimental and control group

Outcome	Estimated mean (SE)		Difference (CI)	Cohen's d / OR (CI)	Difference between medians (CI)	p
	G + T	T				
**MET minutes**	1666 (67)	1620 (65)	46 (−137,228)	0.03 (−0.09, 0.16)	−10 (−221,196)	0.62_mean_ / 0.96_median_
**Alcohol consumption**	1.46 (0.08)	1.34 (0.08)	0.12 (−0.1,0.34)	0.07 (−0.06, 0.20)	< 0.01 (−0.001,0.036)	0.28_mean_ / 0.36_median_
**Vegetable and fruit consumption**	3.51 (0.04)	3.51 (0.04)	−0.001 (−0.1,0.1)	< 0.01 (−0.12, 0.12)	NA	0.99
**Seeking medical treatment/examination (prob.)**	0.09 (0.019)	0.085 (0.019)	0.005 (−0.049,0.059)	1.06 (0.54,2.09)	NA	0.86

Estimated means are marginal means calculated from the associated regression models (marginal mean for the “Seeking medical treatment/examination” is the predicted probability that the participant sought medical treatment or examination). Difference refers to difference between the estimated means. Cohen's d is estimated from the regression model’s t-statistic and degrees of freedom. OR is odds ratio from the logistic regression model. Difference between medians is estimated only for "Alcohol consumption" and "MET minutes". “G+T” (Genetic+Traditional) and “T” (Traditional) are the experimental group and control group. MET minutes are rounded to zero decimal places, and all other variables to 2 decimal places

### Interactive effects of the experimental/control group by T2D or CHD 10-year risk level

No significant interaction between the experimental/control group and the T2D risk level (*p*s > 0.08) or CHD risk level was found (*p*s > 0.26). For most outcomes, the estimated conditional means of the experimental and control group showed relatively similar patterns when inspected in categories of T2D or CHD disease risk. To gauge the interactions' standardized effect size, we used the associated regression models' t-statistic for the interaction term and error degrees of freedom to estimate Cohen's d for the relevant contrast (i.e., the contrast of contrasts). Likewise, we used the interaction term's odd ratio to evaluate the effect of the logistic models. All effects were negligible or small (d = 0.001–0.11, OR = 1.20 for T2D, and d = 0.02–0.07, OR = 1.26 for CHD). See Supplementary File 6 for tables and plots.

### Sensitivity analyses

Since the main analyses were conducted with the assumption that the data was missing completely at random (MCAR) and the exclusion of participants was done on a PP basis, we performed sensitivity analyses with modified ITT type analyses and using MI data.

The data was imputed using all the sociodemographic factors listed in Table 2, relaxing the mechanism of missingness from MCAR to missingness depending on the sociodemographic factors and the model used in MI (see Supplementary File 6 for details). The imputation was done once with PP exclusions and once without any exclusions. Then, the same regression models described above were used to test the mean differences between the experimental and control groups. Finally, we performed the same analyses using the S3 and S4 samples with no exclusions (i.e., ITT with complete cases only). All the analyses led to relatively similar estimates and made no difference regarding the conclusions. The only notable difference was that the imputed models estimated higher probability for participants to seek medical treatment/examination, but the estimates were elevated for both the control and the experimental group (see Supplementary File 7 for complete results).

## Discussion

We conducted a RCT to investigate the effects of supplementing traditional risk estimates of T2D and CHD with PRS-based estimates concerning the same diseases. The experimental group received a 10-year risk estimate based on their PRS and traditional risk, and the control group received a similar estimate based solely on their traditional risk factors. Approximately 80 days later, on average, respondents self-reported physical activity, alcohol consumption, vegetable and fruit consumption, and whether the respondents had sought medical treatment/examination due to the risk scores received were measured.

In general, participants in both groups demonstrated relatively similar lifestyle habits compared to the general population. Over 62% of participants in both groups engaged in endurance exercise consistent with Finnish recommendations, 78% consumed alcohol once a week or less, and more than 52% and 53% consumed vegetables and fruits daily or almost daily, respectively. By comparison, the corresponding estimates for the Finnish population at the time were approximately 50%, 79%, 48%, and 50% (Koponen et al. 2018). We found no significant differences between the groups in relation to any of the outcomes measured. We did not formally test an equivalence range for the effects. However, the confidence intervals indicate relatively modest effects in all cases. The results align with other studies in which active control groups have been used to investigate whether PRS disclosure motivates health behavior change (Hollands et al. 2016; Peterson et al. 2018; King et al. 2023). Similar results were obtained concerning the exploratory analysis, which tested the interaction between T2D or CHD risk level and the experimental/control group. Sensitivity analyses using PP/ITT approaches and imputed data did not change any of the conclusions.

To sum up, we did not find support for the idea that supplementing traditional risk estimates with PRS-based estimates offer additional benefits for health behavioral change. The existence of a minor effect is always possible, but the clinical importance appears doubtful. It seems that simply providing a new type of risk information does not in itself provide a strong incentive for people to make demanding lifestyle changes.

The lack of evidence can be due to a variety of reasons. First, the intervention was not strongly theory-driven in the sense that postulates from theories of health behavior change would have been directly incorporated in the intervention (Marteau and Weinman 2006; Cameron et al. 2017). Instead, we presented the risk information in a way that would likely match a formula that healthcare providers might use. Participants were simply encouraged to engage in behaviors such as exercising more, eating healthier, and maintaining a normal weight. In contrast, a more theory-driven approach might have explicitly linked the presentation of PRS to specific constructs proposed by health behavior theories—such as outcome expectancies, risk perception, or self-efficacy (e.g., Schwarzer and Hamilton 2020). The lack of theory has often been cited as a plausible reason why PRS disclosure has produced muted effects in RCT set-ups (e.g., Driver et al. 2022). However, it remains to be seen if more theory-influenced interventions can display benefits for supplementing more traditional estimates with PRS risk. So far, there is minimal information on how different health behavior change theories work in the context of PRS disclosure.

Second, the effect of receiving PRS estimates may vary between different subgroups. For example, it has been speculated that PRS-based estimates could offer more benefits to younger populations where traditional risk estimates concerning many diseases might still be relatively low (King et al. 2023). Thus, even if the population average effect is close to zero, some specific subgroups might benefit (or be harmed) from receiving PRS in addition to traditional risk estimates. On a related note, further investigation may also be needed into how people react when PRS and traditional risk estimates produce conflicting results (i.e., PRS risk is high and traditional risk is low, or vice versa). In general, fatalism or over-optimism produced by the reception of PRS has not been observed, but studies investigating the effect of explicit discrepancy between different estimates are still largely missing.

Third, various methodological reasons might also contribute to the muted effects. The issues range from the use of self-report measures to biased sample and from the lack of measurement precision to uncertainty about whether the intervention was delivered as intended. We discuss these and other methodological issues more thoroughly in the limitations section. However, it should be noted that most RCTs, using various measures and methodological choices, have produced similar nonsignificant results for PRS disclosure, hinting that methodological issues alone might not exhaustively explain the results.

Furthermore, when discussing the lack of support for an effect on health behavior change, it is also important to note that the conclusion can be drawn both ways. That is, we did not find beneficial effects of supplementing the risk estimates with PRS, but neither did we find detrimental effects. Thus, worries concerning PRS-induced fatalism or over-optimism related to one's disease risk remain unconfirmed (Hunter et al. 2008; Claassen et al. 2010). Given that receiving PRS results has not been found to lead to significant psychosocial distress (e.g., Halmesvaara et al. 2022; Wade 2019) and attitudes toward receiving the estimates have generally been positive (e.g., Leitsalu et al. 2023), it is perhaps not surprising that adverse effects are not seen at the behavioral level either. However, similar caveats concerning the effect heterogeneity apply as described above.

Finally, in addition to the typical health behavior measures, we also investigated if receiving the PRS estimate would increase visits to healthcare providers. As explained previously, a lower threshold for seeking healthcare can be beneficial if there is a need for care, but it can also strain the system if a visit is unnecessary. Since we did not find evidence for an overall difference between the groups or an interaction between the 10-year disease risk level and whether PRS was disclosed or not, it seems that PRS neither provides additional motivation to seek treatment when there might be a need for it (i.e., when the overall disease risk was something else than low) nor does it encourage unnecessary visits to a substantial degree. The results should tentatively allay some concerns about the provision of PRS estimates and the burden on the healthcare system (Sud et al. 2023). However, the above-discussed caveats apply here, too. Moreover, we did not seek to partition all the possible combinations of different disease risk classes (i.e., PRS/overall, low/high, T2D/CHD risk) or the possible contradictions between traditional risk and PRS risk estimate, so a degree of caution should be used when generalizing the results.

### Limitations and future directions

As with any study, several limitations should be noted when interpreting the results. Since actual risk information in a real-world setting was disclosed, the experimental control was less than perfect. We could not precisely control when the participants decided to open their risk estimates and how long it took them to complete the follow-up surveys afterward. As the experimental and control groups showed little difference concerning the time interval between seeing the results and answering the survey questions, and since the time interval did not notably correlate with any of the outcomes, the confounding related to treatment effect should be minor. However, the lack of precise control over response times blurs the interpretation of whether the experimental effect describes a long- or short-term effect (for most respondents, over 75%, it was between 60 and 95 days). On a related note, since the time intervals were obviously measured after the intervention took place and then used as an exclusion criterion in the PP analysis, this might have introduced additional bias in the analysis due to conditioning on a post-treatment variable (Montgomery et al. 2018). Again, given the low correlations with the outcomes and the fact that ITT analysis did not change the conclusions, the bias probably remained relatively small.

Another limitation regarding the trial design was the timing of the S4 survey. Since the proper RCT part of the study ended after S3 (i.e., all participants had access to all risk information) and the seeking treatment/examination was measured at S4, it should be noted that it is theoretically possible that some of the control group participants might have sought treatment/examination based on their PRS results. However, as explained earlier, since the time interval between returning S3 and S4 was merely some hours/minutes (on average, it took 22 min to return the S4 after S3 was completed), it is unlikely that the brief contamination of the control group at the very end of the follow-up had a substantial effect on the results. Still, caution is warranted when generalizing the results. Finally, we did not have manipulation check measures to indicate how closely the respondents scrutinized their results, which adds to the uncertainty regarding how well the intervention was implemented in the experimental and control groups.

In addition to the weakness of the experimental control, the high attrition after randomization is also a significant limitation of the study (approximately 62% for S3 and 87% for S4). Although differential attrition between the experimental and control groups remained relatively low, and neither of the analyses using the imputed data changed the conclusions, caution should still be applied when generalizing results from data with such high attrition (Jakobsen et al. 2017). It is also worth noting that although the participants came from a population-based sample, the current study is not population-based per se but represents volunteers from the FinHealth survey interested in learning about their polygenic risk.

Measurement precision should also be considered when interpreting the results. First, since baseline measurements of the outcomes were unavailable, the accuracy was inevitably lower than in a design where the models would have been adjusted for baseline differences. Second, the measures used were selected based on the FinHealth survey, which is primarily intended for population-based descriptive analysis. The self-report measures used might not have been ideal for an experimental setting. We tried to make the measures as sensitive as possible (as the intervention was relatively subtle) by avoiding categorical coding of the outcome variables when possible. However, it seems likely that the continuous approximations used (especially MET minutes and alcohol consumption) contained at least a moderate amount of measurement error, which could have made the measures less sensitive to detect differences between the groups (i.e., more susceptible to Type-II error; we do not suspect there was a differential measurement error between the groups).

Finally, as discussed earlier, it is important to keep in mind that we did not attempt to model all the possible combinations of different risk types and magnitudes when calculating the results. In the primary analysis, both disease and all risk levels were considered together (i.e., we estimated the treatment effect averaged over all specific disease risk levels). The exploratory analysis, in turn, calculated separate interactions for the overall 10-year risk of T2D or CHD and whether the respondent received a PRS, but it did not explicitly consider the size of the PRS or the three-way interaction between the T2D and CHD risk. Thus, it’s possible that the average effects reported here could mask more complex interactions between different risk types and levels, which should be kept in mind when generalizing the results.

Future studies should pay attention to different types of subgroup analyses of the PRS effect. Subgroups could include different demographic groups but also groups at different levels of disease risk. Attention could also be paid to situations where risk estimates conflict (e.g., lifestyle risk factors and PRS). More explicit use of behavior change theories may also reveal effects that are not captured by current interventions. Finally, when feasible, measures other than self-reporting should be considered to measure health behavior to increase measurement precision and decrease bias.

## Supplementary Information

Below is the link to the electronic supplementary material.

Supplementary file1 (PDF 695 KB)

Supplementary file2 (PDF 765 KB)

Supplementary file3 (PDF 491 KB)

Supplementary file4 (PDF 179 KB)

Supplementary file5 (PDF 163 KB)

Supplementary file6 (PDF 223 KB)

Supplementary file7 (PDF 579 KB)

## Data Availability

The dataset analysed for this study is available to the scientific community based on a written application to the THL Biobank and Finnish biobank legislation. Instructions for submitting an application are provided on the Biobank website (https://thl.fi/en/web/thl-biobank/for-researchers).

## Footnotes

1 https://thl.fi/en/web/thlfi-en/research-and-development/research-and-projects/the-p5.fi-study-genetic-information-for-health-support?redirect=%2Fen%2Fweb%2Fthlfi-en%2Fresearch-and-expertwork%2Fprojects-and-programmes%2Fcurrent-research-and-projects¨

2 It is important to note that even though both the control and experimental group had access to all the risk estimates at S4, the time interval between S3 and S4 was zero days for all participants analyzed, making it highly unlikely that the control group would have had time to seek medical treatment/examination based on the risk scores they had seen some hours/minutes ago (the average interval was approximately 22 min between returning S3 and S4).

3 The S2 measure was intended to serve as a baseline for the health behavior questions. Since the assessment of health behavior concerned at least the last seven days, the assessment should not have been subject to treatment effect if the questionnaire was returned immediately after seeing the risk estimates. Unfortunately, this was not the case, as most returned the questionnaire several days later. Furthermore, there was a large difference between the experimental and control groups in terms of the time taken to return the S2 questionnaire (this was not the case for S3), with valid cases being very unevenly distributed between the groups. For unknown reasons, many respondents (over two hundred) also skipped the S2 survey entirely and went straight to the S3 survey.

4 After the RCT part of the study was over, the VTE risk was supplemented with a PRS-based estimate.

## References

[CR1] Abouzeid M, Wikström K, Peltonen M, Lindström J, Borodulin K, Rahkonen O, Laatikainen T (2015) Secular trends and educational differences in the incidence of type 2 diabetes in Finland, 1972–2007. Eur J Epidemiol 30:649–659. 10.1007/s10654-015-0008-7 25837966 10.1007/s10654-015-0008-7

[CR2] Abul-Husn NS, Kenny EE (2019) Personalized medicine and the power of electronic health records. Cell 177:58–69. 10.1016/j.cell.2019.02.039 30901549 10.1016/j.cell.2019.02.039 PMC6921466

[CR3] Akinshin A (2022) Trimmed Harrell-Davis quantile estimator based on the highest density interval of the given width. Commun Stat - Simul Comput 53:1565–1575. 10.1080/03610918.2022.2050396

[CR4] Baranger DAA, Finsaas MC, Goldstein BL, Vize CE, Lynam DR, Olino TM (2023) Tutorial: power analyses for interaction effects in cross-sectional regressions. Adv Methods Pract Psychol Sci 6:25152459231187532. 10.1177/25152459231187531 10.1177/25152459231187531 PMC12341451 40799847

[CR5] Borodulin K, Sääksjärvi K (2019) FinHealth 2017 Study : methods. https://www.julkari.fi/handle/10024/139084. Accessed 6 Aug 2024

[CR6] Cameron LD, Biesecker BB, Peters E, Taber JM, Klein WMP (2017) Self-regulation principles underlying risk perception and decision making within the context of genomic testing. Soc Personal Psych 11:e12315. 10.1111/spc3.12315 10.1111/spc3.12315 PMC5716481 29225669

[CR7] Claassen L, Henneman L, De Vet R, Knol D, Marteau T, Timmermans D (2010) Fatalistic responses to different types of genetic risk information: exploring the role of self-malleability. Psychol Health 25:183–196. 10.1080/08870440802460434 20391214 10.1080/08870440802460434

[CR8] Croux C, Dhaene G, Hoorelbeke D (2004) Robust standard errors for robust estimators. K.U.Leuven, Faculty of Economics and Applied Economics : Department of Economics. Available VIA Lirias. https://lirias.kuleuven.be/1824142&lang=en. Accessed 21 Aug 2024

[CR9] Driver MN, Kuo SI-C, Dick DM (2022) Returning complex genetic risk information to promote better health-related behaviors: a commentary of the literature and suggested next steps. Transl Behav Med 13:115–119. 10.1093/tbm/ibac071 10.1093/tbm/ibac071 PMC9972341 36125098

[CR10] Dziak JJ, Dierker LC, Abar B (2020) The interpretation of statistical power after the data have been gathered. Curr Psychol 39:870–877. 10.1007/s12144-018-0018-1 32523323 10.1007/s12144-018-0018-1 PMC7286546

[CR11] Faul F, Erdfelder E, Lang A-G, Buchner A (2007) G*Power 3: A flexible statistical power analysis program for the social, behavioral, and biomedical sciences. Behav Res Methods 39:175–191. 10.3758/BF03193146 17695343 10.3758/bf03193146

[CR12] Frieser MJ, Wilson S, Vrieze SI (2018) behavioral impact of return of genetic test results for complex disease: systematic review and meta-analysis. Health Psychol 37:1134–1144. 10.1037/hea0000683 30307272 10.1037/hea0000683 PMC6263735

[CR13] Fuchsberger C, Flannick J, Teslovich TM et al (2016) The genetic architecture of type 2 diabetes. Nature 536:41–47. 10.1038/nature18642 27398621 10.1038/nature18642 PMC5034897

[CR14] Godino JG, van Sluijs EMF, Marteau TM, Sutton S, Sharp SJ, Griffin SJ (2016) Lifestyle advice combined with personalized estimates of genetic or phenotypic risk of type 2 diabetes, and objectively measured physical activity: a randomized controlled trial. PLoS Med 13:e1002185. 10.1371/journal.pmed.1002185 27898672 10.1371/journal.pmed.1002185 PMC5127499

[CR15] Halmesvaara O, Vornanen M, Kääriäinen H, Perola M, Kristiansson K, Konttinen H (2022) Psychosocial effects of receiving genome-wide polygenic risk information concerning type 2 diabetes and coronary heart disease: a randomized controlled trial. Front Genet 13. 10.3389/fgene.2022.881349 10.3389/fgene.2022.881349 PMC9189371 35706448

[CR16] Haskell WL, Lee I-M, Pate RR, Powell KE, Blair SN, Franklin BA, Macera CA, Heath GW, Thompson PD, Bauman A (2007) Physical activity and public health: updated recommendation for adults from the American college of sports medicine and the American heart association. Med Sci Sports Exerc 39:1423–1434. 10.1249/mss.0b013e3180616b27 17762377 10.1249/mss.0b013e3180616b27

[CR17] Hingorani AD, Gratton J, Finan C, Schmidt AF, Patel R, Sofat R, Kuan V, Langenberg C, Hemingway H, Morris JK, Wald NJ (2023) Performance of polygenic risk scores in screening, prediction, and risk stratification: secondary analysis of data in the Polygenic Score Catalog. BMJ Medicine 2. 10.1136/bmjmed-2023-000554 10.1136/bmjmed-2023-000554 PMC10582890 37859783

[CR18] Hoenig JM, Heisey DM (2001) The abuse of power: the pervasive fallacy of power calculations for data analysis. Am Stat 55:19–24. 10.1198/000313001300339897

[CR19] Hollands GJ, French DP, Griffin SJ, Prevost AT, Sutton S, King S, Marteau TM (2016) The impact of communicating genetic risks of disease on risk-reducing health behaviour: systematic review with meta-analysis. BMJ 352:i1102. 10.1136/bmj.i1102 26979548 10.1136/bmj.i1102 PMC4793156

[CR20] Hunter DJ, Khoury MJ, Drazen JM (2008) Letting the genome out of the bottle–will we get our wish? N Engl J Med 358:105–107. 10.1056/NEJMp0708162 18184955 10.1056/NEJMp0708162

[CR21] Jakobsen JC, Gluud C, Wetterslev J, Winkel P (2017) When and how should multiple imputation be used for handling missing data in randomised clinical trials – a practical guide with flowcharts. BMC Med Res Methodol 17:162. 10.1186/s12874-017-0442-1 29207961 10.1186/s12874-017-0442-1 PMC5717805

[CR22] Khera AV, Chaffin M, Aragam KG, Haas ME, Roselli C, Choi SH, Natarajan P, Lander ES, Lubitz SA, Ellinor PT, Kathiresan S (2018) Genome-wide polygenic scores for common diseases identify individuals with risk equivalent to monogenic mutations. Nat Genet 50:1219–1224. 10.1038/s41588-018-0183-z 30104762 10.1038/s41588-018-0183-z PMC6128408

[CR23] King A, Graham CA-M, Glaister M, Da Silva AV, Pilic L, Mavrommatis Y (2023) The efficacy of genotype-based dietary or physical activity advice in changing behavior to reduce the risk of cardiovascular disease, type II diabetes mellitus or obesity: a systematic review and meta-analysis. Nutr Rev 81:1235–1253. 10.1093/nutrit/nuad001 36779907 10.1093/nutrit/nuad001

[CR24] Knowles JW, Zarafshar S, Pavlovic A, Goldstein BA, Tsai S, Li J, McConnell MV, Absher D, Ashley EA, Kiernan M, Ioannidis JPA, Assimes TL (2017) Impact of a genetic risk score for coronary artery disease on reducing cardiovascular risk: a pilot randomized controlled study. Front Cardiovasc Med 4. 10.3389/fcvm.2017.00053 10.3389/fcvm.2017.00053 PMC5558259 28856136

[CR25] Koponen P, Borodulin K, Lundqvist A, Sääksjärvi K, Koskinen S (2018) Terveys, toimintakyky ja hyvinvointi Suomessa: FinTerveys 2017-tutkimus. Available via Julkari https://www.julkari.fi/handle/10024/136223. Accessed 21 Aug 2024

[CR26] Krier JB, Kalia SS, Green RC (2016) Genomic sequencing in clinical practice: applications, challenges, and opportunities. Dialogues Clin Neurosci 18:299–312. 10.31887/DCNS.2016.18.3/jkrier 27757064 10.31887/DCNS.2016.18.3/jkrier PMC5067147

[CR27] Kullo IJ, Jouni H, Austin EE, Brown S-A, Kruisselbrink TM, Isseh IN, Haddad RA, Marroush TS, Shameer K, Olson JE, Broeckel U, Green RC, Schaid DJ, Montori VM, Bailey KR (2016) Incorporating a genetic risk score into coronary heart disease risk estimates: effect on low-density lipoprotein cholesterol levels (the MI-GENES Clinical Trial). Circulation 133:1181–1188. 10.1161/CIRCULATIONAHA.115.020109 26915630 10.1161/CIRCULATIONAHA.115.020109 PMC4803581

[CR28] Leitsalu L, Reigo A, Palover M, Nikopensius T, Läll K, Krebs K, Reisberg S, Mägi R, Kals M, Alavere H, Nõukas M, Kolk A, Normet I, Tammesoo M-L, Käärik E, Puusepp M, Metsalu K, Allik A, Milani L, Fischer K, Tõnisson N, Metspalu A (2023) Lessons learned during the process of reporting individual genomic results to participants of a population-based biobank. Eur J Hum Genet 31:1048–1056. 10.1038/s41431-022-01196-6 36192438 10.1038/s41431-022-01196-6 PMC10474261

[CR29] Marjonen H, Marttila M, Paajanen T, Vornanen M, Brunfeldt M, Joensuu A, Halmesvaara O, Aro K, Alanne-Kinnunen M, Jousilahti P, Borodulin K, Koskinen S, Tuomi T, Ilanne-Parikka P, Lindström J, Laine MK, Auro K, Kääriäinen H, Perola M, Kristiansson K (2021) A web portal for communicating polygenic risk score results for health care use—the P5 study. Front Genet 12. 10.3389/fgene.2021.763159 10.3389/fgene.2021.763159 PMC8585790 34777479

[CR30] Marteau TM, Weinman J (2006) Self-regulation and the behavioural response to DNA risk information: A theoretical analysis and framework for future research. Soc Sci Med 62:1360–1368. 10.1016/j.socscimed.2005.08.005 16162383 10.1016/j.socscimed.2005.08.005

[CR31] Montgomery JM, Nyhan B, Torres M (2018) How conditioning on posttreatment variables can ruin your experiment and what to do about it. Am J Polit Sci 62:760–775. 10.1111/ajps.12357

[CR32] Moorthie S, Martschenko DO, Fatumo S (2023) Are we nearly there yet? Starts and stops on the road to use of polygenic scores. J Community Genet 14:439–440. 10.1007/s12687-023-00672-w 37759103 10.1007/s12687-023-00672-w PMC10576688

[CR33] O’Connell NS, Dai L, Jiang Y, Speiser JL, Ward R, Wei W, Carroll R, Gebregziabher M (2017) Methods for analysis of pre-post data in clinical research: a comparison of five common methods. J Biom Biostat 8:1–8. 10.4172/2155-6180.1000334 30555734 10.4172/2155-6180.1000334 PMC6290914

[CR34] Ong KL, Stafford LK, McLaughlin SA et al (2023) Global, regional, and national burden of diabetes from 1990 to 2021, with projections of prevalence to 2050: a systematic analysis for the Global Burden of Disease Study 2021. The Lancet 402:203–234. 10.1016/S0140-6736(23)01301-6 10.1016/S0140-6736(23)01301-6 PMC10364581 37356446

[CR35] Park JK, Lu CY (2023) Polygenic scores in the direct-to-consumer setting: challenges and opportunities for a new era in consumer genetic testing. J Pers Med 13:573. 10.3390/jpm13040573 37108959 10.3390/jpm13040573 PMC10144199

[CR36] Peterson EB, Chou WS, Gaysynsky A, Krakow M, Elrick A, Khoury MJ, Kaphingst KA (2018) Communication of cancer-related genetic and genomic information: a landscape analysis of reviews. Transl Behav Med 8:59–70. 10.1093/tbm/ibx063 29385592 10.1093/tbm/ibx063 PMC6065548

[CR37] Posit Team (2023) RStudio: Integrated Development Environment for R. Posit Software, PBC, Boston, MA. http://www.posit.co/

[CR38] R Core Team (2023). R: A language and environment for statistical computing. R Foundation for Statistical Computing, Vienna, Austria. https://www.R-project.org/

[CR39] Rainey C, McCaskey K (2021) Estimating logit models with small samples. Polit Sci Res Methods 9:549–564. 10.1017/psrm.2021.9

[CR40] Roberts R, Stewart AFR (2012) Genes and coronary artery disease: where are we? J Am Coll Cardiol 60:1715–1721. 10.1016/j.jacc.2011.12.062 23040572 10.1016/j.jacc.2011.12.062

[CR41] Salomaa V, Pietilä A, Peltonen M, Kuulasmaa K (2016) Changes in CVD incidence and mortality rates, and life expectancy: North Karelia and national. Glob Heart 11:201–205. 10.1016/j.gheart.2016.04.005 27242087 10.1016/j.gheart.2016.04.005

[CR42] Sanchis-Gomar F, Perez-Quilis C, Leischik R, Lucia A (2016) Epidemiology of coronary heart disease and acute coronary syndrome. Ann Transl Med 4:256. 10.21037/atm.2016.06.33 27500157 10.21037/atm.2016.06.33 PMC4958723

[CR43] Schwarzer R, Hamilton K (2020) Changing Behavior Using the Health Action Process Approach. In: Hagger MS, Cameron LD, Hamilton K, Hankonen N, Lintunen T (eds) The Handbook of Behavior Change, 1st edn. Cambridge University Press, pp 89–103

[CR44] Sud A, Horton RH, Hingorani AD, Tzoulaki I, Turnbull C, Houlston RS, Lucassen A (2023) Realistic expectations are key to realising the benefits of polygenic scores. BMJ 380:e073149. 10.1136/bmj-2022-073149 36854461 10.1136/bmj-2022-073149 PMC9973128

[CR45] Vaduganathan M, Mensah GA, Turco JV, Fuster V, Roth GA (2022) The Global Burden of Cardiovascular Diseases and Risk. J Am Coll Cardiol 80:2361–2371. 10.1016/j.jacc.2022.11.005 36368511 10.1016/j.jacc.2022.11.005

[CR46] Viigimaa M, Jürisson M, Pisarev H, Kalda R, Alavere H, Irs A, Saar A, Fischer K, Läll K, Kruuv-Käo K, Mars N, Widen E, Ripatti S, Metspalu A (2022) Effectiveness and feasibility of cardiovascular disease personalized prevention on high polygenic risk score subjects: a randomized controlled pilot study. Eur Heart J Open 2:oeac079. 10.1093/ehjopen/oeac079 36600884 10.1093/ehjopen/oeac079 PMC9803971

[CR47] Voils CI, Coffman CJ, Grubber JM, Edelman D, Sadeghpour A, Maciejewski ML, Bolton J, Cho A, Ginsburg GS, Yancy WS (2015) Does type 2 diabetes genetic testing and counseling reduce modifiable risk factors? a randomized controlled trial of veterans. J Gen Intern Med 30:1591–1598. 10.1007/s11606-015-3315-5 25876740 10.1007/s11606-015-3315-5 PMC4617940

[CR48] Wade CH (2019) What is the psychosocial impact of providing genetic and genomic health information to individuals? an overview of systematic reviews. Hastings Center Report 49. 10.1002/hast.1021 10.1002/hast.1021 31268566

[CR49] Wallingford CK, Kovilpillai H, Jacobs C, Turbitt E, Primiero CA, Young M-A, Brockman DG, Soyer HP, McInerney-Leo AM, Yanes T (2023) Models of communication for polygenic scores and associated psychosocial and behavioral effects on recipients: a systematic review. Genet Med 25:1–11. 10.1016/j.gim.2022.09.008 36322150 10.1016/j.gim.2022.09.008

[CR50] Wang P-Y, Fang J-C, Gao Z-H, Zhang C, Xie S-Y (2016) Higher intake of fruits, vegetables or their fiber reduces the risk of type 2 diabetes: a meta-analysis. J Diabetes Investig 7:56–69. 10.1111/jdi.12376 26816602 10.1111/jdi.12376 PMC4718092

[CR51] Widén E, Junna N, Ruotsalainen S, Surakka I, Mars N, Ripatti P, Partanen JJ, Aro J, Mustonen P, Tuomi T, Palotie A, Salomaa V, Kaprio J, Partanen J, Hotakainen K, Pöllänen P, Ripatti S (2022) How Communicating Polygenic and Clinical Risk for Atherosclerotic Cardiovascular Disease Impacts Health Behavior: an Observational Follow-up Study. Circ: Genomic Prec Med 15:e003459. 10.1161/CIRCGEN.121.003459 10.1161/CIRCGEN.121.003459 35130028

[CR52] Wilcox RR (2022) Introduction to robust estimation and hypothesis testing, Fifth edition. Academic Press, an imprint of Elsevier, London, United Kingdom San Diego, United States Cambridge, MA Oxford, United Kingdom

[CR53] Zheng Y, Ley SH, Hu FB (2018) Global aetiology and epidemiology of type 2 diabetes mellitus and its complications. Nat Rev Endocrinol 14:88–98. 10.1038/nrendo.2017.151 29219149 10.1038/nrendo.2017.151

[CR54] Zurbau A, Au-Yeung F, Blanco Mejia S, Khan TA, Vuksan V, Jovanovski E, Leiter LA, Kendall CWC, Jenkins DJA, Sievenpiper JL (2020) Relation of different fruit and vegetable sources with incident cardiovascular outcomes: a systematic review and meta-analysis of prospective cohort studies. J Am Heart Assoc 9:e017728. 10.1161/JAHA.120.017728 33000670 10.1161/JAHA.120.017728 PMC7792377

