# Supplementary material for: The impact of supplementing traditional risk information with polygenic risk score concerning type 2 diabetes and coronary heart disease on health behavior: a randomized controlled trial
Source: J Community Genet. 2025 Mar 26;16(3):373–86. doi: 10.1007/s12687-025-00790-7 (PMC12202269; doi:10.1007/s12687-025-00790-7)
Supplement: Supplementary file 1 — Supplementary file1 (PDF 695 KB) [file 12687_2025_790_MOESM1_ESM.pdf]

Journal of Community Genetics

The Impact of Supplementing Traditional Risk Information with Polygenic Risk Score Concerning Type 2 Diabetes and Coronary Heart Disease on Health Behavior: A Randomized Controlled Trial

Otto Halmesvaara[1*], Marleena Lonna [2,3], Helena Kääriäinen[3], Markus Perola[2,3], Kati Kristiansson[2,3], Hanna Konttinen[1]

[1] Social Psychology, Faculty of Social Sciences, University of Helsinki, Helsinki, Finland

[2] Research Program for Clinical and Molecular Metabolism, Faculty of Medicine, University of Helsinki, Helsinki, Finland

[3] Department of Public Health, Finnish Institute for Health and Welfare, Helsinki, Finland

*** Correspondence:**

Otto Halmesvaara

otto.halmesvaara@helsinki.fi

Supplementary File 1

Figure 1. Return dates for S1, S3, and S4

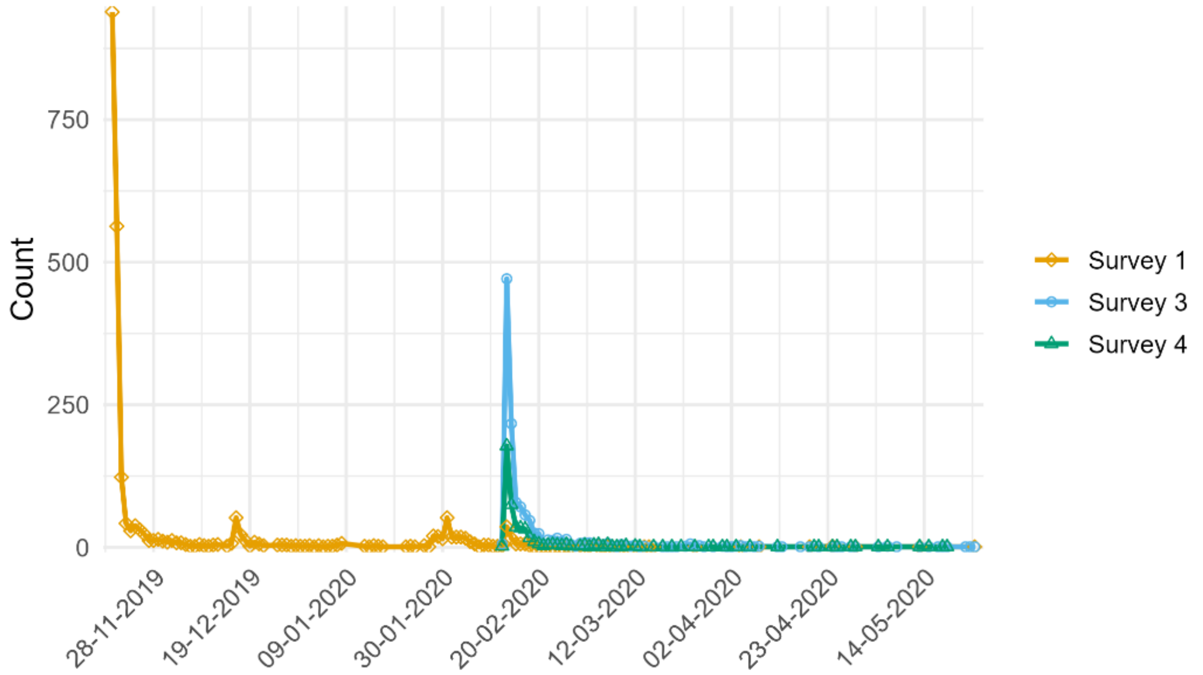

Figure 2. S1-3 and S1-4 time interval (in days) between the experimental and control group

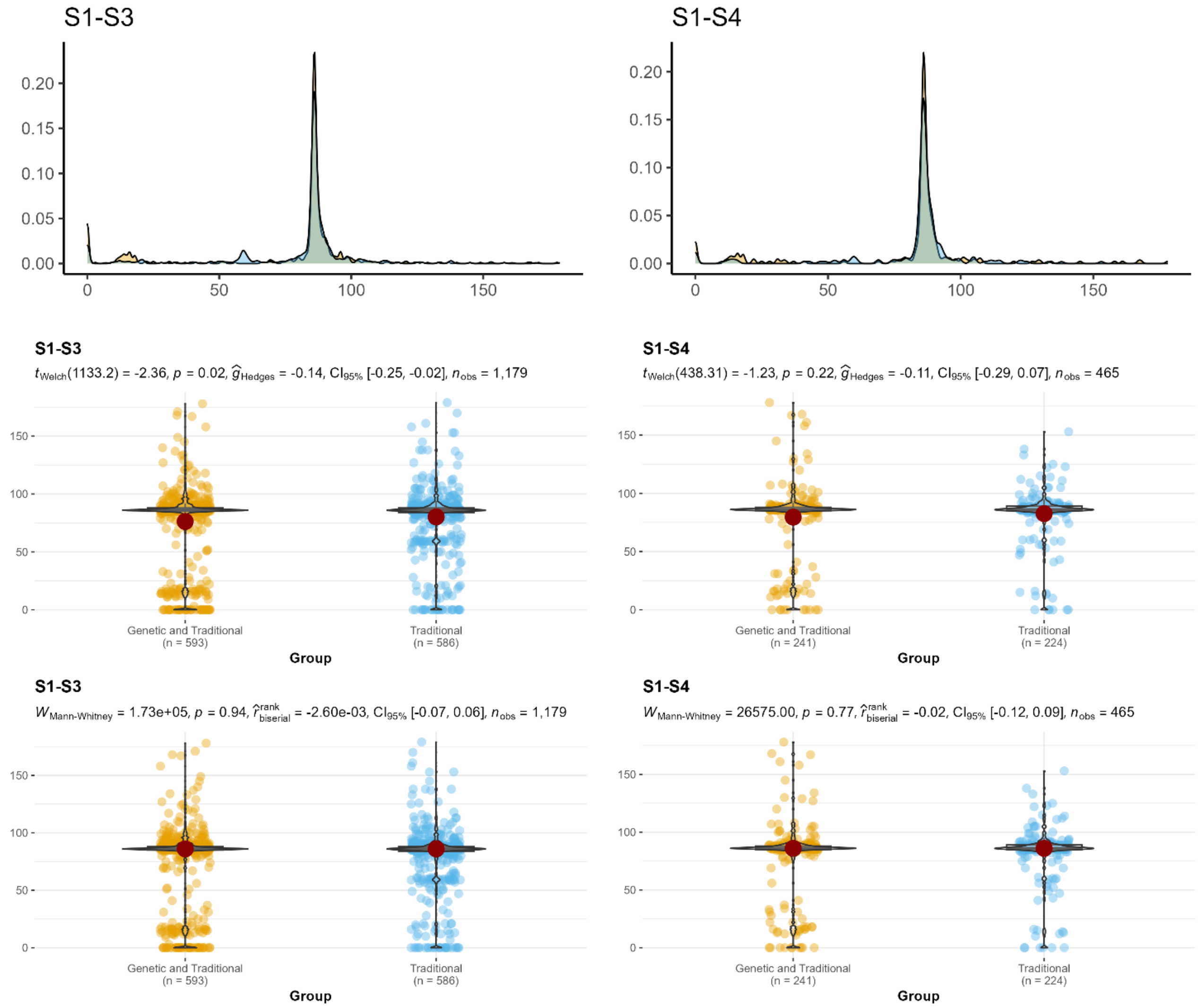

As can be seen from Figure 2, the time interval between S1-3 and S1-4 differed only slightly between the groups. The means were very close to each other (S1-3: 76.26_{g+t} vs. 80.22_{t}; S1-4: 79.57_{g+t} vs. 82.60_{t}), and the medians and modes were identical (S1-3: 86; S1-4: 86). Moreover, the quartiles also matched closely (.25 quantile = $85_{g+t(s1\text{-}3)}$ vs $84_{t(s1\text{-}3)}$ / $85_{g+t(s1\text{-}4)}$ vs $85_{t(s1\text{-}4)}$ and .75 quantile = $88_{g+t(s1\text{-}3)}$ vs $88_{t(s1\text{-}3)}$ / $88_{g+t(s1\text{-}4)}$ vs $89_{t(s1\text{-}4)}$). At the level of the whole data, 50 % of the respondents completed both S3 and S4 85-88 days after seeing their results, and over 75 % of the respondents between 60-95 days after seeing their results.

Table 1. Correlation between S1-3 and S1-4 time interval and the outcome measures

Var		r	p	τ	p	n
S1-3 int. & S3 outcome	MET minutes	-.037	.206	-.019	.525	1161
	Alcohol	.033	.265	.007	.8	1149
	Vegetable/fruit	.029	.326	.015	.601	1179

S1-4 int. & sought treatment S4	.032	.489	-.009	.853	465

Note. Int. refer to time interval (in days) respondent had between filling in particular surveys. r is Pearson's r and tau is Kendall's tau.

Figure 3. LOESS-regression between time interval (S1-S3 or S1-S4) and the outcome measures

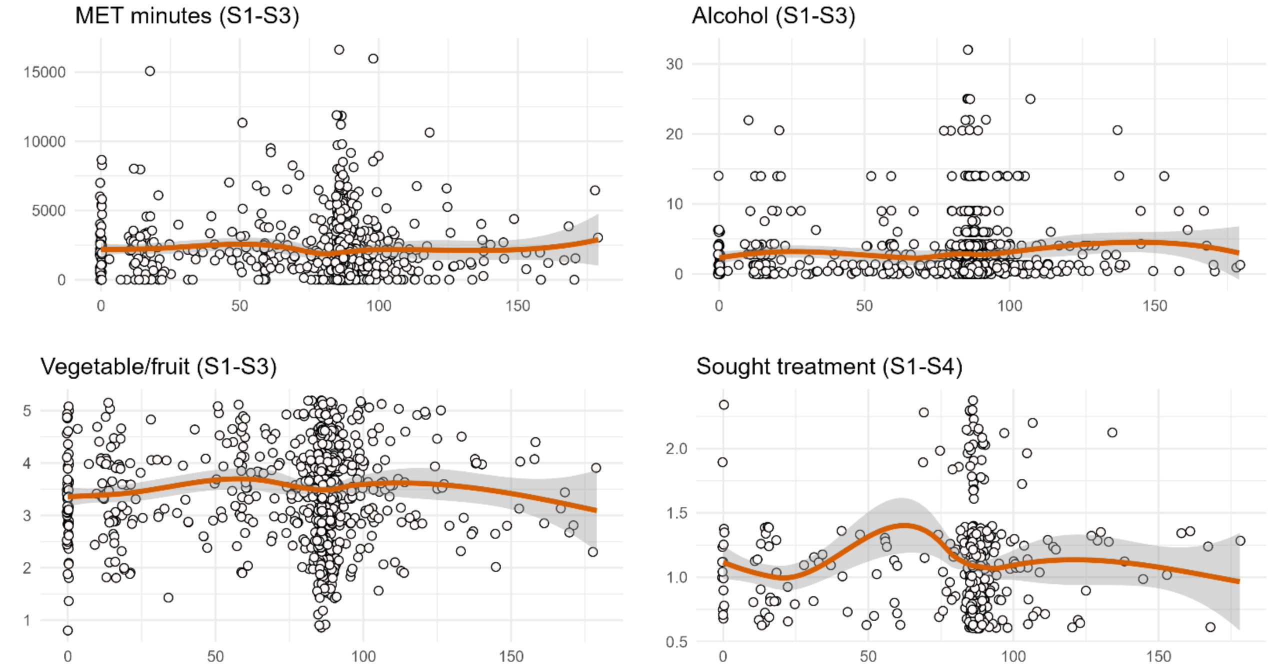

As Table 1 and Figure 3 show, the time between seeing the results (S1) and returning the outcome measure survey (S3/S4) did not notably correlate with any of the outcomes. The LOESS-regression (using the base R loess() function with default settings) between S1-S4 and the sought treatment measure does show curvature around 25-75 days. However, as so few data points exist before day 86, the regression is highly susceptible to individual "outliers." Consequently, the curvature reflects the responses of just 3 participants whose time interval was between 69 and 75 days and who reported that they sought treatment/examination.

Exclusions and Sample Characteristics

Table 2 in the main article presents respondents' sociodemographic characteristics after randomization and in the two surveys used (i.e., S3 and S4). As can be seen from the table, compared to the initial randomized sample (i.e., respondents who agreed to participate in the P5 study and had sufficient data to be included in the randomization; n = 3177), there were fewer respondents in S3 and S4 from the oldest age groups (over 70 y.), people with only comprehensive education, pensioners, people with an annual income of 25000 € or less, and people at high risk for

CHD. Conversely, S3 and S4 surveys included more middle-aged respondents (30-60), people with higher education, people who are employed, people with an annual income of more than 80000 €, and people at low risk for CHD. Attrition within the experimental group and within the control group was, in general, reasonably similar. The highest differences were in the S4 sample, where the control group had fewer participants with intermediate education (25.2 vs. 33.5 %), more respondents with higher education (66.4 vs. 60 %), and fewer employed participants (56.2 vs. 63.3 %).

Figure 1 in the main article provides a general breakdown of response rates for each survey used in the primary analysis and the deletions concerning each model per experimental/control group. For the PP analysis, we excluded participants from all models with single clinical variants (SCV) relating to CHD or T2D, T2D/CHD already diagnosed, or who did not return the S1 survey. Moreover, we excluded participants from models estimating PA and alcohol consumption if the respondent's S1-3 time interval was less than 21 days (since the questions were asked in relation to the last three weeks). Likewise, in the model estimating vegetable and fruit consumption, we excluded respondents with S1-3 time interval of less than seven days (for the same reasons). Finally, when estimating the probability that the respondents sought medical treatment/examination, we excluded respondents if their S1-4 time interval was less than ten days (which was the smallest number after zero)[1].

[1] We wanted the S4 measure point to reflect the treatment effect as closely as possible. If the respondents had returned S1, S3, and S4 on the same day, they would only have had some hours/minutes to seek treatment/examination before answering the S4 (the average interval was approximately 22 minutes between returning S3 and S4). Moreover, it would have been difficult to argue that the control group's results would not reflect the PRS results they received after returning S3 (compared to a situation where the S1-S4 interval was longer and the control group thus had access only to their traditional estimates for the overwhelming majority of the follow-up).
