# Supplementary material for: The impact of supplementing traditional risk information with polygenic risk score concerning type 2 diabetes and coronary heart disease on health behavior: a randomized controlled trial
Source: J Community Genet. 2025 Mar 26;16(3):373–86. doi: 10.1007/s12687-025-00790-7 (PMC12202269; doi:10.1007/s12687-025-00790-7)
Supplement: Supplementary file 3 — Supplementary file3 (PDF 491 KB) [file 12687_2025_790_MOESM3_ESM.pdf]

Journal of Community Genetics

The Impact of Supplementing Traditional Risk Information with Polygenic Risk Score Concerning Type 2 Diabetes and Coronary Heart Disease on Health Behavior: A Randomized Controlled Trial

Otto Halmesvaara[1*], Marleena Lonna [2,3], Helena Kääriäinen[3], Markus Perola[2,3], Kati Kristiansson[2,3], Hanna Konttinen[1]

[1] Social Psychology, Faculty of Social Sciences, University of Helsinki, Helsinki, Finland

[2] Research Program for Clinical and Molecular Metabolism, Faculty of Medicine, University of Helsinki, Helsinki, Finland

[3] Department of Public Health, Finnish Institute for Health and Welfare, Helsinki, Finland

*** Correspondence:**

Otto Halmesvaara

otto.halmesvaara@helsinki.fi

Supplementary File 3

Outcome measures

MET minutes

Average MET minutes per week were estimated based on the following question: "*Overall, how physically active are you during the week at work, on your ways to and from work, and during your leisure time? Think about the previous three weeks. Take into account all regular weekly physical activity which lasts at least 10 minutes per session.*", where participants marked different minutes and hours for "*Calm and low-intensity physical activity (= does not make you sweat or get out of breath, e.g. slow walking)", "Brisk and vigorous physical activity (= makes you sweat a bit and/or get slightly out of breath, e.g. brisk walking)", and "High-intensity aerobic physical activity (= makes you sweat a lot and/or get out of breath, e.g. jogging or running)".* Participants could also indicate that they had "*Hardly any regular activity each week".*

If the respondent marked "Hardly any regular activity" or had less than 10 minutes of combined PA activity, MET minutes were set to zero. Moreover, we screened the responses for implausibly high values of overall PA minutes per week. At face value, we estimated that if the combined PA time of all activity types (low-intensity PA, brisk and vigorous PA, high-intensity aerobic PA) averaged 12 hours or more per day (12*60*7 = 5040 min per week), it was likely due to misreporting of actual PA and was assigned as missing value (n=2).

After implausible values were set to missing, MET minutes were calculated for each PA type and multiplied by suitable MET value. MET values were obtained from approximations suggested by Haskell et al. (2007), where common low-intensity PA activities have a MET coefficient of 1.5-3, moderate-intensity activities have a MET coefficient of 3-6, and high-intensity activities have a MET coefficient of 6-11.5. For low-intensity and moderate-intensity PA, we used the midpoint of the suggested range. However, given the higher range in suggested MET values for high-intensity activities, we decided to be more conservative and use the lower quartile (25th percentile) as an estimate. Thus, the selected approximations for MET values were 2.25 for low-intensity activities, 4.5 for moderate, and 7.375 for high-intensity PA.

Figure 1. Counts for different PA items (in minutes) and for the subsequent MET variables

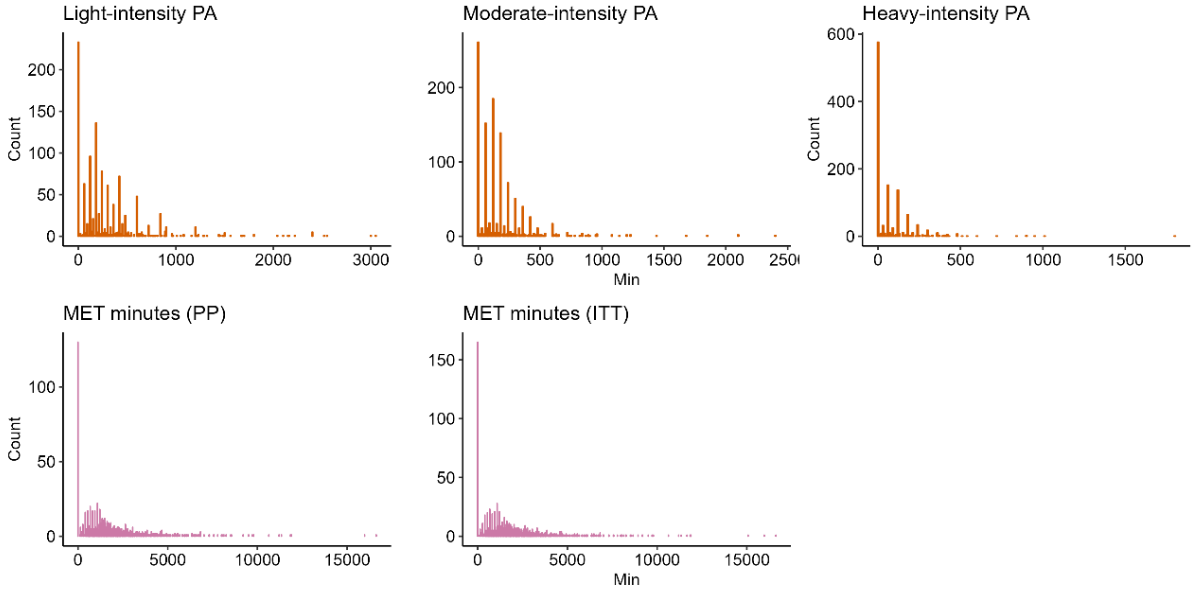

Note. PP = sample used in per-protocol analysis and ITT = sample used in intention-to-treat analysis

Alcohol consumption

The average alcohol consumption per week was estimated using two questions. 1) "*How often do you drink alcoholic beverages? (1=Never, 2=Once a month or less frequently, 3=2–4 times a month, 4=2–3 times a week, 5=4 times or more a week)*" and 2) "*How many drinks containing alcohol have you drunk on a typical day when you have been drinking? (1=1–2 servings, 2=3–4 servings, 3=5–6 servings, 4=7–9 servings, 5=10 or more servings)*" Servings were defined using the Finnish definition of 12 grams of pure alcohol per serving (THL). Different examples were given to the respondents to illustrate how the alcohol portion is defined (e.g., "*0.33 l medium-strength beer, cider or long drink, max 4.7%*").

Then, to obtain a continuous measure of average portions per week, values from 1) and 2) were combined. Weekly drinking frequencies were estimated as the midpoint of the reported range (standardized as per week portions when needed): 1 = 0, 2=0.25, 3=0.75, 4=2.5, 5=4. Similarly, for drinking rates, the midpoint of the reported range was used: 1=1.5, 2=3.5, 3=5.5, 4=8, 5=10. Finally, the value of drinking frequencies was multiplied by the value of drinking rates. For example, 2-4 drinking times per month with 5-6 servings per occasion would translate into 0.75*5.5= 4.125 portions per week.

Figure 2. Counts for different alcohol items and for the subsequent average alcohol consumption (per w.) variables

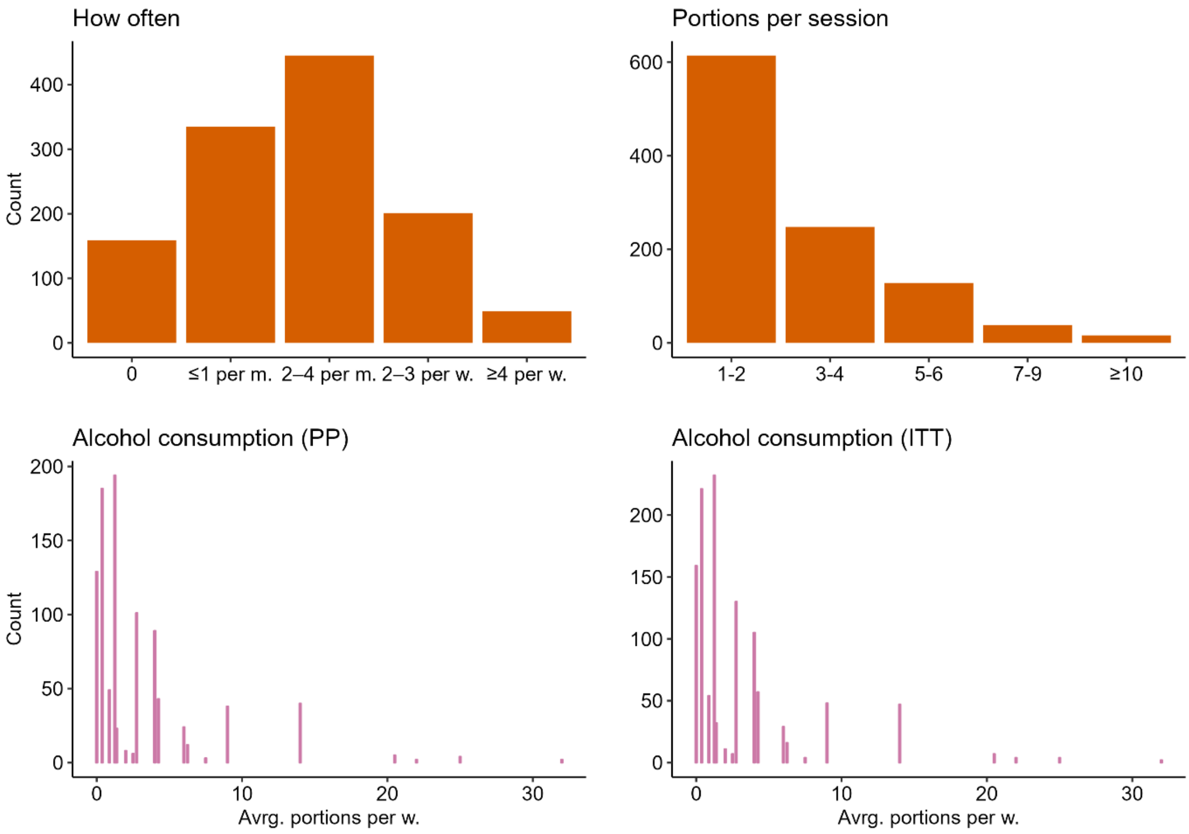

Note. PP = sample used in per-protocol analysis and ITT = sample used in intention-to-treat analysis

Vegetable and fruit consumption

Vegetable and fruit/berries consumption was measured using two questions. 1) "How often have you eaten fruit or berries within the past 7 days?" and 2) "How often have you eaten vegetables (not including potatoes) within the previous 7 days as such, grated or in a fresh salad?". Both had similar response scales: "1=Not at all, 2=1 to 2, 3=3 to 5 days, 4=on 6 to 7 days, 5=Several times a day". As both vegetable and fruit consumption are similarly related to T2D and CHD risk (Wang et al., 2016; Zurbau et al., 2020) and showed a reasonable correlation with each other (r=.44; alpha=.61), we created a simple composite score that used the mean of the two variables.

Figure 3. Counts for different vegetable and fruit items and for the subsequent composite score

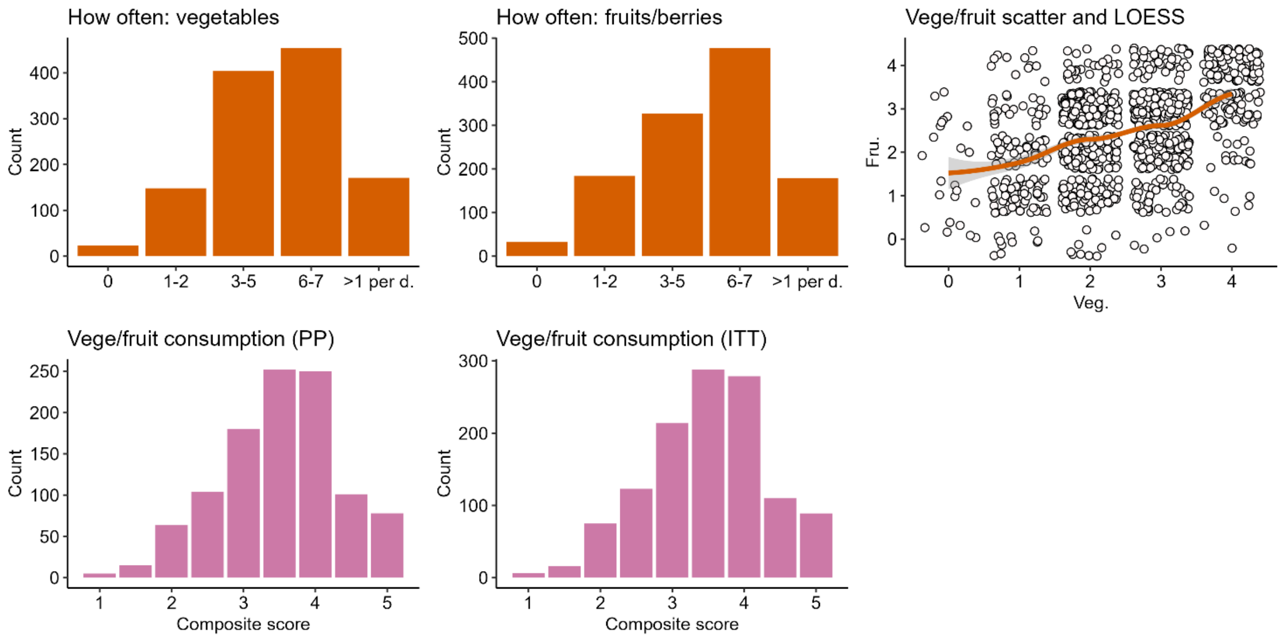

Note. PP = sample used in per-protocol analysis and ITT = sample used in intention-to-treat analysis

Did the participants sought medical treatment/examination

Whether the participant had sought medical treatment/examination was measured with one question: "Have you sought a medical examination or treatment (with a physician, a public health nurse or a nurse) based on the feedback you received from the P5 study? (1=No, I have not, 2=I might do that, 3=Yes, I have)". Since we were interested only in actual behavior and due to the previously mentioned issues with the S4 measure point, we classified participants who answered that they might seek medical treatment as "No" answers (n = 13).

Figure 4. Counts for PP and ITT samples concerning whether the respondent sought medical treatment/examination

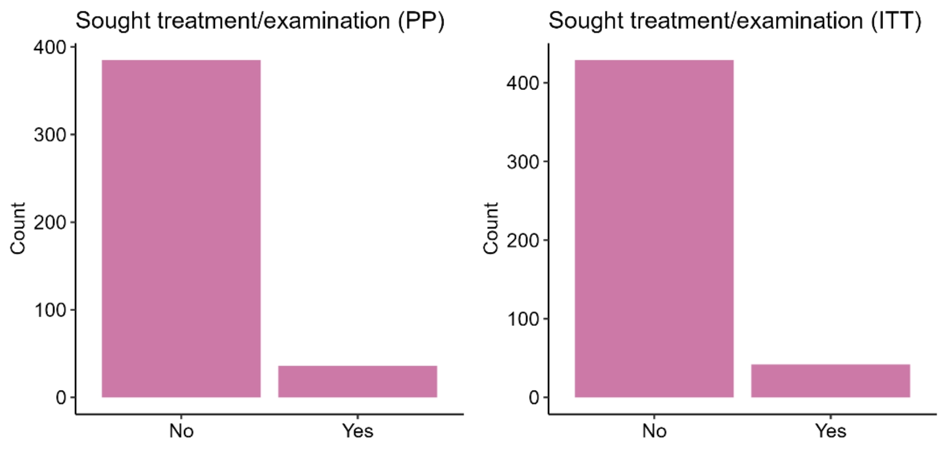

Note. PP = sample used in per-protocol analysis and ITT = sample used in intention-to-treat analysis

References

Haskell WL, Lee I-M, Pate RR, Powell KE, Blair SN, Franklin BA, Macera CA, Heath GW, Thompson PD, Bauman A (2007) Physical activity and public health: updated recommendation for adults from the American College of Sports Medicine and the American Heart Association. Med Sci Sports Exerc 39:1423–1434. https://doi.org/10.1249/mss.0b013e3180616b27

Wang P-Y, Fang J-C, Gao Z-H, Zhang C, Xie S-Y (2016) Higher intake of fruits, vegetables or their fiber reduces the risk of type 2 diabetes: A meta-analysis. J Diabetes Investig 7:56–69. https://doi.org/10.1111/jdi.12376

Zurbau A, Au-Yeung F, Blanco Mejia S, Khan TA, Vuksan V, Jovanovski E, Leiter LA, Kendall CWC, Jenkins DJA, Sievenpiper JL (2020) Relation of Different Fruit and Vegetable Sources With Incident Cardiovascular Outcomes: A Systematic Review and Meta-Analysis of Prospective Cohort Studies. J Am Heart Assoc 9:e017728. https://doi.org/10.1161/JAHA.120.017728
