# Supplementary material for: The impact of supplementing traditional risk information with polygenic risk score concerning type 2 diabetes and coronary heart disease on health behavior: a randomized controlled trial
Source: J Community Genet. 2025 Mar 26;16(3):373–86. doi: 10.1007/s12687-025-00790-7 (PMC12202269; doi:10.1007/s12687-025-00790-7)
Supplement: Supplementary file 4 — Supplementary file4 (PDF 179 KB) [file 12687_2025_790_MOESM4_ESM.pdf]

Journal of Community Genetics

The Impact of Supplementing Traditional Risk Information with Polygenic Risk Score Concerning Type 2 Diabetes and Coronary Heart Disease on Health Behavior: A Randomized Controlled Trial

Otto Halmesvaara[1*], Marleena Lonna [2,3], Helena Kääriäinen[3], Markus Perola[2,3], Kati Kristiansson[2,3], Hanna Konttinen[1]

[1] Social Psychology, Faculty of Social Sciences, University of Helsinki, Helsinki, Finland

[2] Research Program for Clinical and Molecular Metabolism, Faculty of Medicine, University of Helsinki, Helsinki, Finland

[3] Department of Public Health, Finnish Institute for Health and Welfare, Helsinki, Finland

*** Correspondence:**

Otto Halmesvaara

otto.halmesvaara@helsinki.fi

Supplementary File 4

R packages

Analysis	Function	Package
Imputation	mice(), pool()	mice (Van Buuren & Groothuis-Oudshoorn, 2011)
Logistic regression (all)	glm() / brglmFit()	base R / brglm2 (Kosmidis & Firth, 2021)
Marginal means	emmeans(), contrast() / avg_predictions(), avg_comparisons()	emmeans (Lenth, 2023) / marginaleffects (Arel-Bundock, 2024)
Median difference	qcomhd()	WRS2 (Mair & Wilcox, 2020)
OLS regression (all)	lm()	base R
Plots	ggplot_the_model() / ggplot() / plot_predictions()	custom function by Jeffrey Walker (Walker, 2020) / ggplot2 (Wickham, 2016) / marginaleffects (Arel-Bundock, 2024)
Power analysis (interaction)	power_interaction()	InteractionPoweR (Baranger et al., 2023)
Robust regression (interaction)	lmrob()	robustbase (Maechler et al., 2024)
Robust regression (primary)	rlm()	MASS (Venables & Ripley, 2002)
Standardized effect size	t_to_d(), r_to_d(), r_to_oddsratio()	effectsize (Ben-Shachar et al., 2020)

References

Arel-Bundock V (2024) marginaleffects: Predictions, Comparisons, Slopes, Marginal Means, and Hypothesis Tests. R package version 0.20.1, https://CRAN.R-project.org/package=marginaleffects.

Baranger DAA, Finsaas MC, Goldstein BL, Vize CE, Lynam DR, Olino TM (2023) Tutorial: Power Analyses for Interaction Effects in Cross-Sectional Regressions. Advances in Methods and Practices in Psychological Science 6:25152459231187531. https://doi.org/10.1177/25152459231187531

Ben-Shachar MS, Lüdecke D, Makowski D (2020) effectsize: Estimation of Effect Size Indices and Standardized Parameters. Journal of Open Source Software 5:2815. https://doi.org/10.21105/joss.02815

Buuren S van, Groothuis-Oudshoorn K (2011) mice: Multivariate Imputation by Chained Equations in R. Journal of Statistical Software 45:1–67. https://doi.org/10.18637/jss.v045.i03

Kosmidis I, Firth D (2021) Jeffreys-prior penalty, finiteness and shrinkage in binomial-response generalized linear models. Biometrika 108:71–82. https://doi.org/10.1093/biomet/asaa052

Lenth, R (2023) emmeans: Estimated Marginal Means, aka Least-Squares Means. R package version 1.9.0, https://CRAN.R-project.org/package=emmeans

Mair P, Wilcox R (2020) Robust statistical methods in R using the WRS2 package. Behav Res 52:464–488. https://doi.org/10.3758/s13428-019-01246-w

Maechler M, Rousseeuw P, Croux C, Todorov V, Ruckstuhl A, Salibian-Barrera M, Verbeke T, Koller M, Conceicao EL, Anna di Palma M (2024) robustbase: Basic Robust Statistics. R package version 0.99-2, http://robustbase.r-forge.r-project.org

Venables WN, Ripley BD (2002) Modern Applied Statistics with S. Springer New York, New York, NY

Walker JA (2020) Elements of Statistical Modeling for Experimental Biology. Unpublished manuscript.

Wickham H (2009) ggplot2: Elegant Graphics for Data Analysis. Springer New York, New York, NY
