# Supplementary material for: The impact of supplementing traditional risk information with polygenic risk score concerning type 2 diabetes and coronary heart disease on health behavior: a randomized controlled trial
Source: J Community Genet. 2025 Mar 26;16(3):373–86. doi: 10.1007/s12687-025-00790-7 (PMC12202269; doi:10.1007/s12687-025-00790-7)
Supplement: Supplementary file 5 — Supplementary file5 (PDF 163 KB) [file 12687_2025_790_MOESM5_ESM.pdf]

Journal of Community Genetics

The Impact of Supplementing Traditional Risk Information with Polygenic Risk Score Concerning Type 2 Diabetes and Coronary Heart Disease on Health Behavior: A Randomized Controlled Trial

Otto Halmesvaara[1*], Marleena Lonna [2,3], Helena Kääriäinen[3], Markus Perola[2,3], Kati Kristiansson[2,3], Hanna Konttinen[1]

[1] Social Psychology, Faculty of Social Sciences, University of Helsinki, Helsinki, Finland

[2] Research Program for Clinical and Molecular Metabolism, Faculty of Medicine, University of Helsinki, Helsinki, Finland

[3] Department of Public Health, Finnish Institute for Health and Welfare, Helsinki, Finland

*** Correspondence:**

Otto Halmesvaara

otto.halmesvaara@helsinki.fi

Supplementary File 5

Power

Table 1. Sensitivity power calculations with g*power for pp primary analysis

Outcome	Sample per group	Test	Effect size at 80 % power
**MET minutes**	474/500	T-test	d = .180
**Alcohol**	459/498	T-test	d = .181
**Vegetable & fruit**	525/526	T-test	d = .173
**Seeking treatment**	216/205	Large sample z-test (Wald)	OR = 2.2

Note. OR refer to odds ratio and d to Cohen's d. Alpha is set to .05 in all tests (two tailed). T-test is independent means t-test and assumes equal variances. Large sample z-test is based on Demidenko (2007) paper. Binomial distribution is spesified for treatment variable, sample is assumed to be balanced, Pr(Y=1 | X=1) under the null is assumed to be .09. Difference in probabilities was Pr(Y=1 | X=1) H1 = .185 (Δ = .095).

Table 2. Sensitivity power for the PP interaction effects using correlation-based power simulations

Outcome	Risk	Effect size at 80 % power
**MET minutes**	T2D	r = .14
	CHD	r = .15
**Alcohol**	T2D	r = .14
	CHD	r = .15
**Vegetable & fruit**	T2D	r = .14
	CHD	r = .14
**Seeking treatment**	T2D	r = .27
	CHD	r = .27

Note. r = Pearson's r. Simulations were done with InteractionPoweR (Baranger et al., 2023) package. Excluding the correlation between the interaction term and the outcome (which was probed at different levels of r), all correlations between the predictors and between the predictors and the outcome were derived directly from the data. Since the dichotomization of the variables outside the experimental/control group division was artificial, we did not use the correlation adjustment to specify the correct correlation structure. 1000 simulations were used for each effect.

References

Baranger DAA, Finsaas MC, Goldstein BL, Vize CE, Lynam DR, Olino TM (2023) Tutorial: Power Analyses for Interaction Effects in Cross-Sectional Regressions. Advances in Methods and Practices in Psychological Science 6:25152459231187531. https://doi.org/10.1177/25152459231187531

Demidenko E (2007) Sample size determination for logistic regression revisited. Statistics in Medicine 26:3385–3397. https://doi.org/10.1002/sim.2771
