# Supplementary material for: The impact of supplementing traditional risk information with polygenic risk score concerning type 2 diabetes and coronary heart disease on health behavior: a randomized controlled trial
Source: J Community Genet. 2025 Mar 26;16(3):373–86. doi: 10.1007/s12687-025-00790-7 (PMC12202269; doi:10.1007/s12687-025-00790-7)
Supplement: Supplementary file 6 — Supplementary file6 (PDF 223 KB) [file 12687_2025_790_MOESM6_ESM.pdf]

Journal of Community Genetics

The Impact of Supplementing Traditional Risk Information with Polygenic Risk Score Concerning Type 2 Diabetes and Coronary Heart Disease on Health Behavior: A Randomized Controlled Trial

Otto Halmesvaara[1*], Marleena Lonna [2,3], Helena Kääriäinen[3], Markus Perola[2,3], Kati Kristiansson[2,3], Hanna Konttinen[1]

[1] Social Psychology, Faculty of Social Sciences, University of Helsinki, Helsinki, Finland

[2] Research Program for Clinical and Molecular Metabolism, Faculty of Medicine, University of Helsinki, Helsinki, Finland

[3] Department of Public Health, Finnish Institute for Health and Welfare, Helsinki, Finland

*** Correspondence:**

Otto Halmesvaara

otto.halmesvaara@helsinki.fi

Supplementary File 6

Interactions for PP analysis

Figure 1. Estimated conditional means and 95 % CI for PP models with treatment/control interaction by risk level (complete cases only)

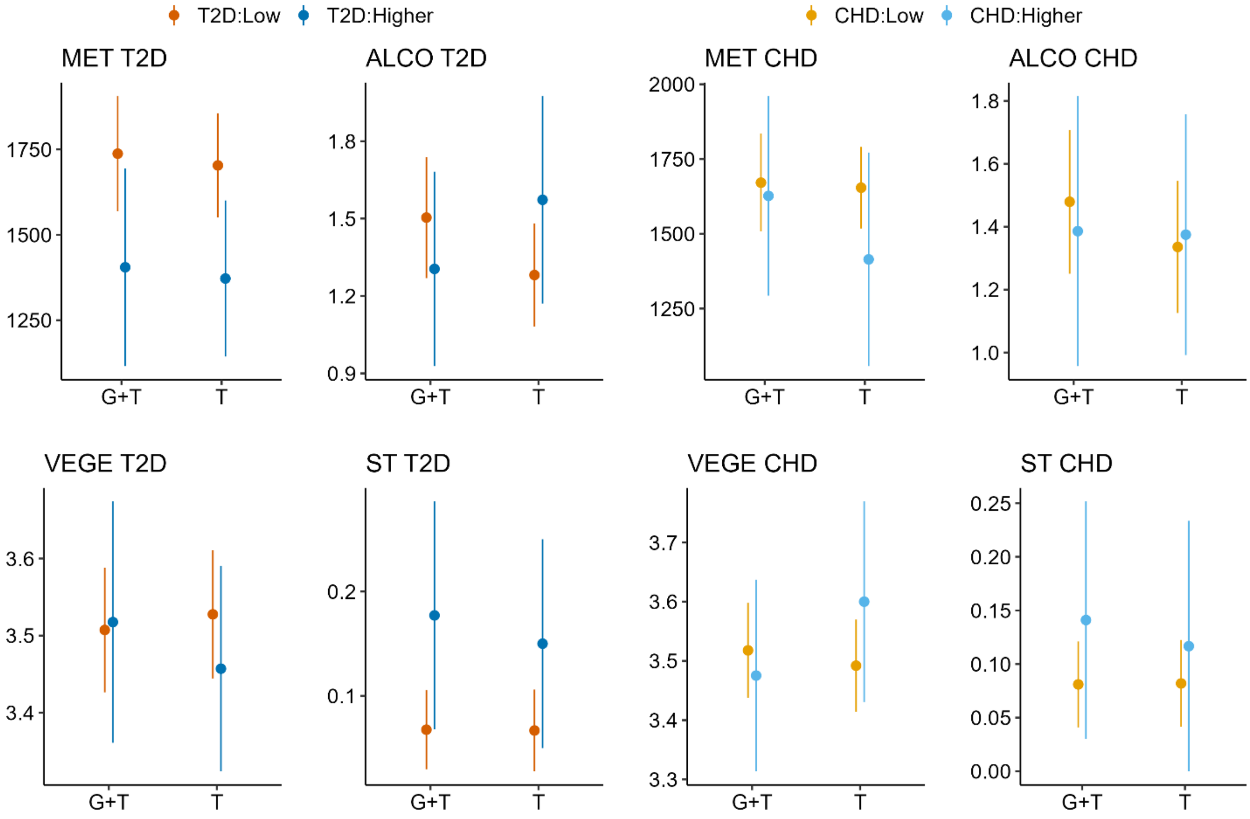

Note. “MET” = average MET minutes (per w.), “ALCO” = average alcohol portions (per w.), “VEGE” = composite score for vegetable and fruit consumption, “ST” = did the participant seek medical treatment/examination after seeing the P5 results (probability), “Low risk” = less than 7.5 % risk of developing T2D/CHD during the next ten years, and “High risk” = more than 7.5 % risk of developing T2D/CHD during the next ten years. G+T = Genetic and traditional (experimental group), and T = Traditional (control group).

As Figure 1 shows, the interaction effects between the groups tend to be, for most variables, very faint. The most substantial effect was found concerning T2D risk and alcohol consumption. Here, the conditional means show signs of crossover interaction, but the differences are tiny. Moreover, given the noisiness of the measure, not much can be said about the pattern with confidence.

Table 1 gives the exact estimates for means, standard errors, p-values, and effect sizes for all models.

Table 1. Estimated conditional means, p-values for interaction term, and estimated effect sizes

Outcome	Risk level	Estimated mean (SE)		Interaction p	Cohen's d/OR
		G+T	T		
**MET minutes (T2D)**	Low	1737 (86)	1703 (78)	0.99	<0.001
	Higher	1405 (148)	1372 (117)		
**MET minutes (CHD)**	Low	1671 (83)	1654 (70)	0.46	-0.048
	Higher	1627 (170)	1415 (182)		
**Alcohol (T2D)**	Low	1.5 (0.12)	1.28 (0.1)	0.08	0.113
	Higher	1.3 (0.19)	1.57 (0.21)		
**Alcohol (CHD)**	Low	1.48 (0.12)	1.34 (0.11)	0.66	0.029
	Higher	1.39 (0.22)	1.37 (0.2)		
**Vegetable (T2D)**	Low	3.51 (0.04)	3.53 (0.04)	0.51	-0.041
	Higher	3.52 (0.08)	3.46 (0.07)		
**Vegetable (CHD)**	Low	3.52 (0.04)	3.49 (0.04)	0.26	0.07
	Higher	3.48 (0.08)	3.6 (0.09)		
**Sought treatment (T2D)**	Low	0.07 (0.02)	0.07 (0.02)	0.79	1.20 (OR)
	Higher	0.18 (0.06)	0.15 (0.05)		
**Sought treatment (CHD)**	Low	0.08 (0.02)	0.08 (0.02)	0.78	1.26 (OR)
	Higher	0.14 (0.06)	0.12 (0.06)		

Note. Heteroscedasticity-consistent HC3 standard errors were used for the vegetable/fruit consumption and robust standard errors by Croux et al., 2003 for the robust regression models. For the non-logistic regression models, the effect sizes were estimated based on the t-statistic of the interaction term and the model degrees of freedom. For the logistic models, the effect size is the odds ratio of the interaction term. MET minutes are rounded to zero decimal places, and all other variables to 2 decimal places.

References

Croux C, Dhaene G, Hoorelbeke D. (2003) Robust standard errors for robust estimators. Discussion Papers Series 03.16, K.U. Leuven, CES.
