# Supplementary material for: The impact of supplementing traditional risk information with polygenic risk score concerning type 2 diabetes and coronary heart disease on health behavior: a randomized controlled trial
Source: J Community Genet. 2025 Mar 26;16(3):373–86. doi: 10.1007/s12687-025-00790-7 (PMC12202269; doi:10.1007/s12687-025-00790-7)
Supplement: Supplementary file 7 — Supplementary file7 (PDF 579 KB) [file 12687_2025_790_MOESM7_ESM.pdf]

Journal of Community Genetics

The Impact of Supplementing Traditional Risk Information with Polygenic Risk Score Concerning Type 2 Diabetes and Coronary Heart Disease on Health Behavior: A Randomized Controlled Trial

Otto Halmesvaara[1*], Marleena Lonna[2,3], Helena Kääriäinen[3], Markus Perola[2,3], Kati Kristiansson[2,3], Hanna Konttinen[1]

[1] Social Psychology, Faculty of Social Sciences, University of Helsinki, Helsinki, Finland

[2] Research Program for Clinical and Molecular Metabolism, Faculty of Medicine, University of Helsinki, Helsinki, Finland

[3] Department of Public Health, Finnish Institute for Health and Welfare, Helsinki, Finland

*** Correspondence:**

Otto Halmesvaara

otto.halmesvaara@helsinki.fi

Supplementary File 7

Modified ITT analysis (complete cases only)

Descriptives

Table 1. Descriptives for ITT analysis

Outcome	n		mean		sd		median		range	
	G+T	T	G+T	T	G+T	T	G+T	T	G+T	T
**MET minutes**	601	581	2025	2076	1830	2195	1620	1611	11662	16620
**Alcohol consumption**	590	579	2.83	2.84	4.06	4.07	1.25	1.25	32	25
**Vegetable and fruit consumption**	609	591	3.49	3.5	0.84	0.82	3.5	3.5	4	4
**Sought treatment/examination**	245	226	**G+T Yes/No**: 24/221;			**T Yes/No**: 18/208				

Note. "G+T" refers to the experimental group, and "T" to the control group. Since "Sought treatment" is a binary variable, proportions in absolute numbers are presented instead of typical descriptive statistics. MET minutes are rounded to zero decimal places, and all other variables to 2 decimal places.

Interactions

Table 2. Estimated conditional means, p-values for interaction term, and estimated effect sizes for ITT analysis

Outcome	Risk level	Estimated mean (SE)		Interaction p	Cohen's d/OR
		G+T	T		
**MET minutes (T2D)**	Low	1798 (77)	1728 (75)	0.9	0.008
	Higher	1388 (119)	1341 (98)		
**MET minutes (CHD)**	Low	1729 (74)	1654 (67)	0.83	-0.012
	Higher	1558 (139)	1439 (138)		
**Alcohol (T2D)**	Low	1.5 (0.11)	1.33 (0.1)	0.34	0.056
	Higher	1.51 (0.17)	1.58 (0.17)		
**Alcohol (CHD)**	Low	1.48 (0.1)	1.37 (0.1)	0.86	-0.011
	Higher	1.63 (0.21)	1.48 (0.19)		
**Vegetable (T2D)**	Low	3.51 (0.04)	3.52 (0.04)	0.65	-0.026
	Higher	3.45 (0.07)	3.43 (0.06)		
**Vegetable (CHD)**	Low	3.51 (0.04)	3.49 (0.04)	0.24	0.068
	Higher	3.41 (0.07)	3.52 (0.07)		
**Sought treatment (T2D)**	Low	0.07 (0.02)	0.06 (0.02)	0.6	1.41 (OR)
	Higher	0.19 (0.05)	0.13 (0.04)		
**Sought treatment (CHD)**	Low	0.08 (0.02)	0.08 (0.02)	0.48	1.68 (OR)
	Higher	0.17 (0.05)	0.11 (0.05)		

Note. “Low risk” = less than 7.5 % risk of developing T2D/CHD during the next ten years, and “High risk” = more than 7.5 % risk of developing T2D/CHD during the next ten years. G+T = Genetic and traditional (experimental group), and T = Traditional (control group). Heteroscedasticity-consistent HC3 standard errors were used for the vegetable/fruit consumption and robust standard errors by Croux et al., 2003 for the robust regression models. For the non-logistic regression models, the effect sizes were estimated based on the t-statistic of the interaction term and the model degrees of freedom. For the logistic models, the effect size is the odds ratio of the interaction term. MET minutes are rounded to zero decimal places, and all other variables to 2 decimal places.

Figure 1. Estimated conditional means and 95 % CI for ITT models with treatment/control interaction by risk level (complete cases only)

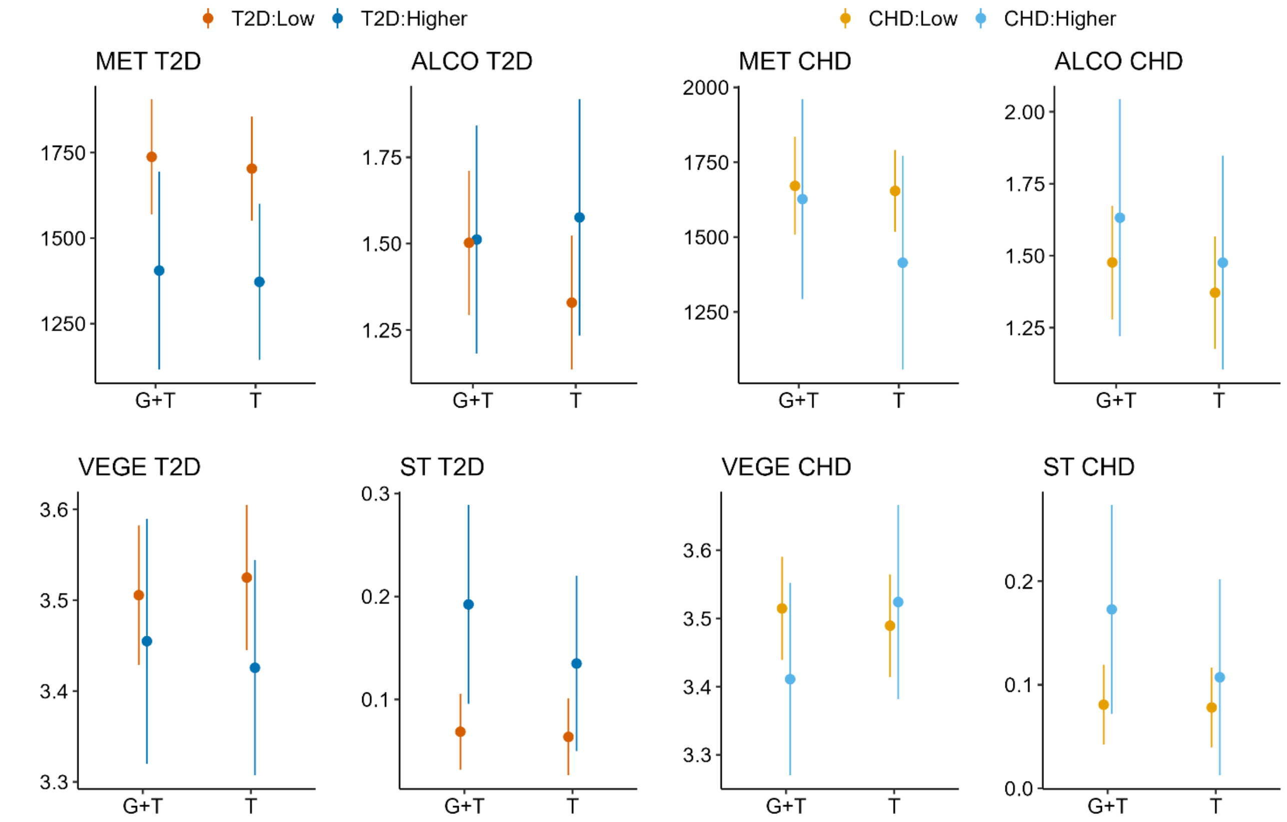

Note. “MET” = average MET minutes (per w.), “ALCO” = average alcohol portions (per w.), “VEGE” = composite score for vegetable and fruit consumption, “ST” = did the participant seek medical treatment/examination after seeing the P5 results (probability), “Low risk” = less than 7.5 % risk of developing T2D/CHD during the next ten years, and “High risk” = more than 7.5 % risk of developing T2D/CHD during the next ten years. G+T = Genetic and traditional (experimental group), and T = Traditional (control group).

Results for primary analysis

Table 3. Estimated marginal means and effect sizes with 95 % CI for ITT analysis

Outcome	Estimated mean (SE)		Difference (CI)	Cohen's d / OR (CI)	Difference between medians (CI)	p
	G+T	T				
**MET minutes**	1695 (59)	1614 (60)	81 (-85,247)	0.06 (-0.06,0.17)	30 (-162,217)	.34$_{mean}$ / .78$_{median}$
**Alcohol**	1.5 (0.07)	1.39 (0.07)	0.11 (-0.09,0.31)	-0.07 (-0.05,0.18)	<0.01 (-0.001,0.039)	.27$_{mean}$ / .29$_{median}$
**Vegetable/fruit**	3.49 (0.03)	3.5 (0.03)	-0.001 (-0.1,0.09)	<0.01 (-0.12,0.11)	NA	.93
**Sought treatment (prob.)**	0.1 (0.019)	0.081 (0.018)	0.018 (-0.034,0.07)	1.25 (0.66,2.35)	NA	.50

Note. The predicted group in "Sought treatment" is that the participant sought medical treatment or examination. Estimated means are marginal means calculated from the associated regression models. Difference refers to difference between the estimated means. Cohen's d is estimated from the regression model’s t-statistic and degrees of freedom. OR is odds ratio from the logistic regression model. Difference between medians is estimated only for "Alcohol" and "MET minutes". G+T and T are the treatment group (Genetic and Traditional risk received) and control group (only Traditional risk received). MET minutes are rounded to zero decimal places, and all other variables to 2 decimal places.

The effects obtained without exclusions were highly similar to those obtained in the PP analysis. Here, the results from the primary analysis produced slightly higher mean differences but less pronounced effects concerning the medians. The interaction effects were, in general, in a similar range or fainter (i.e., closer to zero), excluding the sought treatment variable, where the ITT showed a slightly more pronounced differences compared to PP. However, given the large standard errors for most variables, slight differences in the results are unsurprising and do not change any of the conclusions made from the PP analysis.

Imputation

Imputation was performed with the r-package MICE (Van Buuren & Groothuis-Oudshoorn, 2011) using chained equations (or fully conditional specification), which imputes multivariate missing data on a variable-by-variable basis (Van Buuren, 2021). Imputation was carried out separately by the randomized group (i.e., treatment/control) to allow treatment effect heterogeneity and to make the analysis slightly more robust against plausible interactions by the treatment/control group (Sullivan et al., 2018; Li & Stuart, 2019). Likewise, separate imputations were made using the PP data (i.e., data with some of the reported outcomes set as missing due to improper time interval, diagnosed T2D/CHD, SCV finding, etc.) as a starting point and using intention-to-treat data (i.e., data without exclusions) as a starting point.

A similar setup was used in all imputations. All sociodemographic variables and the T2D/CHD risk were used to predict each outcome variable. Outcome variables and the sociodemographic variables were used to predict each sociodemographic variable. Outcome variables were also used to predict other outcomes in order to preserve the relations between the outcome variables as much as possible. Predictive mean matching was used for all outcomes, and polytomous/binary logistic regression was used for the sociodemographic variables (excluding age, which was entered as a continuous variable in the models, and predictive mean matching was used).

Below are the percentages of missingness per variable, the correlations between the outcome variables, the amount of variance each sociodemographic variable explains for each outcome, and the association between each predictor and missingness.

Table 4. Percentage of missing cases per variable

	PP	ITT
**Treatment/control**	0	0

**Gender**	0	0
**Age**	<0.1	<0.1
**Educational level**	0.3	0.3
**Occupational status**	0.2	0.2
**Annual income**	2.2	2.2
**Marital status**	0.2	0.2
**Has no children**	1.7	1.7
**CHD risk**	0	0
**T2D risk**	0	0
**MET minutes**	69.3	62.8
**Alcohol**	69.9	63.2
**Vegetable/fruit**	67	62.2
**Sought treatment**	86.7	85.2

Note. PP refers to per-protocol and ITT to intention-to-treat

Table 5. Correlations (r) between the outcome variables

	PP				ITT			
	**MET**	**Alcohol**	**V/f**	**St**	**MET**	**Alcohol**	**V/f**	**St**
**MET**								
**Alcohol**	-.01				-.03			
**V/f**	.09*	-.11*			.08*	-.11*		
**St**	-.01	-.09	-.04		-.01	-.09	-.04	

Note. * = p < .05. PP refers to per-protocol and ITT to intention-to-treat. MET = MET minutes; V/f = vegetable/fruit; St = Sought treatment. Correlations are Pearson correlations.

Table 6. Variance explained (R2) when each outcome is regressed on each predictor

	PP				ITT			
	**MET**	**Alcohol**	**V/f**	**St**	**MET**	**Alcohol**	**V/f**	**St**
**Gender**	.007*	.062*	.031*	.003	.005*	.066*	.035*	<.001
**Age**	.002	<.001	.015*	.022*	<.001	<.001	.011*	.021*
**Educational level**	<.001	.002	.024*	.006	.001	.001	.028*	.007
**Occupational status**	.007	.007	.012*	.018	.004	.004	.009*	.018
**Annual income**	.003	.005	.011*	.019	.003	.004	.012*	.016
**Marital status**	.001	<.001	.007*	.005	.001	.001	.006*	.006
**Has no children**	<.001	<.001	.003	.011*	<.001	<.001	.002	.010*
**CHD risk**	<.001	<.001	<.001	.004	<.001	.002	<.001	.009
**T2D risk**	.011*	.005	.004	.023*	.010*	.004	.004	.029*

Note. * = p < .05 for F-test. PP refers to per-protocol and ITT to intention-to-treat. MET = MET minutes; V/f = vegetable/fruit; St = Sought treatment. Regression is estimated with OLS. Model with Sought treatment was calculated as linear probability model (LPM).

Table 7. Association (Cramer's V) between each predictor and missingness in each outcome

	PP				ITT			
	**MET**	**Alcohol**	**V/f**	**St**	**MET**	**Alcohol**	**V/f**	**St**
**Treatment/control**	.017	.026	.001	.011	.014	.008	.012	.017

**Gender**	.032	.034	.031	.038*	.002	.003	.001	.023
**Age group**	.193*	.194*	.207*	.124*	.204*	.205*	.21*	.122*
**Education level**	.159*	.155*	.166*	.117*	.151*	.146*	.154*	.108*
**Occupational status**	.177*	.179*	.188*	.097*	.173*	.175*	.178*	.081*
**Annual income**	.136*	.136*	.145*	.119*	.148*	.148*	.153*	.116*
**Marital status**	.054*	.054*	.057*	.045*	.057*	.056*	.06*	.039
**Has no children**	.04*	.046*	.042*	.044*	.033*	.041*	.037*	.045*
**T2D risk**	.109*	.112*	.118*	.066*	.061*	.061*	.064*	.035
**CHD risk**	.163*	.163*	.172*	.117*	.132*	.134*	.138*	.086*

Note. * = p < .05 for chi-square test. PP refers to per-protocol and ITT to intention-to-treat. MET = MET minutes; V/f = vegetable/fruit; St = Sought treatment.

Table 8. Relative proportion of cases per socio-demographic variable in the models used and missing observations

Variable	Category	PP							
		MET minutes		Alcohol		Vegetable & fruit		Sought treatment	
		% in sample	% in missing	% in sample	% in missing	% in sample	% in missing	% in sample	% in missing
**Treatment/control (%)**	Treatment	48.7	50.5	48	50.8	49.9	50	51.3	49.7
**Gender (%)**	Women	58.4	55	58.6	54.9	58.2	54.9	60.8	55.3
**Age Group (%)**	<30	7.1	6.6	7	6.6	7.4	6.4	5	7
	30-39	17.1	11.8	17.3	11.8	17	11.7	17.1	12.9
	40-49	19.1	13.6	19.2	13.6	19.3	13.3	18.5	14.8
	50-59	25.7	20	26	19.9	25.8	19.7	28	20.8
	60-69	23.5	26.7	22.8	27	23.1	27.1	24	26
	70-79	6.9	17.4	7	17.3	6.8	17.8	7.1	15.2
	80>	0.6	3.9	0.6	3.9	0.7	4	0.2	3.3
**Educational level (%)**	Comprehensive	7.9	18.4	7.9	18.3	7.9	18.8	6.2	16.6
	Intermediate	30	34.1	30.2	34	30.2	34.1	29	33.4
	Higher	62.1	47.5	61.9	47.7	61.8	47.1	64.8	50
**Occupational status (%)**	Employed	63.8	47.2	63.9	47.3	63.8	46.6	61.8	50.9
	Unemployed	5	4.7	5	4.7	5.1	4.7	6.4	4.6
	Pensioner	23.6	41.9	23.3	41.9	23.6	42.6	24.7	38.1
	Student	3.7	3.1	4	3	3.8	3	3.1	3.3
	Other	3.8	3.1	3.8	3.1	3.7	3.1	4	3.2
**Annual income (%)**	25000 € or less	13.2	21.7	13.3	21.6	13.1	22.1	12.5	20.1
	25001 - 45000	23.4	28	23.4	28	23.5	28.1	19.9	27.6
	45001 - 60000	20.1	18.2	19.7	18.4	20.1	18.1	23.3	18.1
	60001 - 80000	20	16.2	19.7	16.4	19.9	16.1	17.5	17.4
	Over 80000 €	23.4	15.9	23.9	15.7	23.4	15.6	26.9	16.8
**Marital status (%)**	Has a partner	75.3	72.8	75.2	72.9	75.7	72.5	75.4	73.3
	Divorcee or widow	12.2	16.2	12.3	16.2	12.1	16.4	11.2	15.6
	Single	12.4	10.9	12.6	10.9	12.1	11	13.4	11.1

**Has no children (%)**		23.9	20.4	24.3	20.2	23.9	20.2	26.1	20.7
**T2D risk (%)**	Low	81.8	66	82	66	81.8	65.5	84.1	68.8
	Elevated	5.6	8.4	5.4	8.4	5.7	8.4	5.7	7.8
	High	12.5	25.6	12.5	25.5	12.5	26.1	10.2	23.4
	Very high	76.7	66.5	77.1	66.4	76.9	66	77.2	68.4
**CHD risk (%)**	Low	7.1	8.2	6.9	8.2	7.1	8.2	6.4	8.1
	Elevated	10.9	15.5	10.6	15.6	10.6	15.8	10.9	14.6
	High	5.3	9.9	5.4	9.8	5.4	10	5.5	9

Continuation

Variable	Category	ITT							
		MET minutes		Alcohol		Vegetable & fruit		Sought treatment	
		% in sample	% in missing	% in sample	% in missing	% in sample	% in missing	% in sample	% in missing
**Treatment/control (%)**	Treatment	50.8	49.4	50.5	49.7	50.7	49.5	52	49.6
**Gender (%)**	Women	55.9	56.1	56.2	55.9	56.1	56	58.8	55.5
**Age Group (%)**	<30	6.9	6.6	6.9	6.6	7.1	6.5	5.1	7
	30-39	16.2	11.8	16.4	11.7	16.4	11.6	16.1	13
	40-49	18.5	13.3	18.6	13.4	18.5	13.3	17.8	14.8
	50-59	25.6	19.4	25.8	19.3	25.8	19.3	27.4	20.7
	60-69	24.6	26.4	24.2	26.7	24.3	26.6	25.9	25.7
	70-79	7.4	18.2	7.4	18.1	7.2	18.4	7.4	15.3
	80>	0.7	4.2	0.7	4.2	0.7	4.3	0.2	3.4
**Educational level (%)**	Comprehensive	9.3	18.7	9.4	18.6	9.3	18.8	7.4	16.5
	Intermediate	30.3	34.4	30.5	34.2	30.3	34.4	29.5	33.4
	Higher	60.4	47	60.1	47.2	60.5	46.8	63.1	50
**Occupational status (%)**	Employed	62.1	46.5	62.2	46.5	62.1	46.3	59.9	51
	Unemployed	4.9	4.8	4.9	4.8	4.9	4.8	5.9	4.6
	Pensioner	25.7	42.6	25.4	42.6	25.4	42.9	27.2	37.9
	Student	3.7	3	3.9	2.9	3.8	2.9	3.2	3.3
	Other	3.6	3.1	3.5	3.2	3.7	3.1	3.8	3.2
**Annual income (%)**	25000 € or less	14	22.1	14.1	22	13.8	22.3	13.7	20
	25001 - 45000	22.9	28.8	22.9	28.7	22.9	28.8	19.5	27.8
	45001 - 60000	19.9	18.2	19.6	18.3	20	18	22.9	18.1
	60001 - 80000	20.5	15.5	20.3	15.7	20.3	15.6	18	17.3
	Over 80000 €	22.8	15.4	23.2	15.3	23	15.2	25.9	16.8
**Marital status (%)**	Has a partner	75.6	72.4	75.5	72.5	75.8	72.3	75.1	73.3
	Divorcee or widow	12.4	16.6	12.4	16.5	12.3	16.7	11.9	15.5
	Single	12	11	12.1	11	11.9	11.1	13	11.1
**Has no children (%)**		23.2	20.4	23.7	20.2	23.4	20.3	25.9	20.7

**T2D risk (%)**	Low	78.6	66.3	78.8	66.2	78.8	66	79.8	69.3
	Elevated	6	8.4	5.9	8.5	5.9	8.5	6.6	7.7
	High	15.4	25.3	15.3	25.3	15.2	25.5	13.6	23
	Very high	72.8	67.7	73.1	67.6	73.1	67.5	73.2	69
**CHD risk (%)**	Low	7.8	7.9	7.5	8	7.7	7.9	7.4	7.9
	Elevated	12.4	15.1	12.4	15	12.2	15.2	11.7	14.5
	High	7	9.4	7	9.4	7	9.4	7.6	8.6

Note. The numbers are the relative proportions of respondents in relation to a given category within a given sample. "% in sample" refers to the sample used to model a particular outcome, and "% in missing" refers to missing observations for that outcome. For example, the numbers 58.6 and 54.9 in the "Alcohol" column and "Gender" row of the first table mean that, for the sample used in the per-protocol analyses to model average alcohol consumption, 58.6% of the respondents in the sample were women, and 54.9% of the missing observations were women (i.e., respondents who were randomized but who for one reason or another did not end up in the alcohol model). PP refers to per-protocol and ITT to intention-to-treat.

We used the highest number of missing cases (~88 % in the sought treatment model for per-protocol analysis using the control group) as a rough indicator of the data sets (m) needed for the imputations. According to Van Buuren and Groothuis-Oudshoorn (2011), typically, a low number of iterations (M = 10-20) is enough to reach convergence. Thus, we set m = 88 and M = 15. After imputation, we inspected the trace plots for signs of non-convergence, and density and box plots for signs of unusual imputed values. When no apparent problems were detected (plots not shown here) and no issues were noted in the logged events, we continued to calculate the sensitivity models with the imputed data.

Imputation results

Table 9. Estimated marginal means and mean differences with 95 % CI for the imputed PP and ITT analysis

Outcome	PP imputation				ITT imputation			
	Estimated mean (SE)		Difference (CI)	p	Estimated mean (SE)		Difference (CI)	p
	G+T	T			G+T	T		
**MET**	1634 (85)	1596 (61)	38 (-166,242)	.71	1691 (60)	1616 (55)	75 (-85,235)	.36
**Alcohol**	1.45 (0.09)	1.32 (0.1)	0.13 (-0.06,0.33)	.18	1.46 (0.07)	1.35 (0.09)	0.11 (-0.08,0.3)	.25
**V/f**	3.49 (0.04)	3.49 (0.04)	<0.01 (-0.12,0.13)	.95	3.49 (0.04)	3.47 (0.04)	0.01 (-0.09,0.11)	.78
**St**	0.16 (0.03)	0.12 (0.03)	0.04 (-0.06,0.14)	.43	0.14 (0.02)	0.11 (0.03)	0.03 (-0.05,0.11)	.49

Note. **OR for PP St = 1.41** and for **ITT St = 1.30**. PP refers to per-protocol and ITT to intention-to-treat. MET = MET minutes; V/f = vegetable/fruit; St = Sought treatment. MET minutes are rounded to zero decimal places, and all other variables to 2 decimal places.

Table 10. Regression coefficient for the interaction term and associated p-value for the imputed PP and ITT analysis of treatment/control group by risk level interaction

Outcome	Risk	PP<br>Interaction coef/OR (CI)	p	ITT<br>Interaction coef/OR (CI)	p
**MET**	T2D	25 (-380,430)	0.9	75 (-261,411)	0.66

	CHD	-75 (-525,375)	0.74	97 (-278,473)	0.61
**Alcohol**	T2D	0.3 (-0.11,0.71)	0.15	0.21 (-0.15,0.58)	0.25
	CHD	0.16 (-0.28,0.6)	0.48	-0.02 (-0.39,0.36)	0.94
**V/f**	T2D	-0.02 (-0.25,0.21)	0.89	-0.02 (-0.24,0.2)	0.87
	CHD	0.13 (-0.1,0.35)	0.26	0.13 (-0.09,0.35)	0.25
**St**	T2D	1.33 (0.33,5.37)	0.69	1.1 (0.39,3.06)	0.86
	CHD	1.05 (0.26,4.21)	0.94	0.91 (0.23,3.6)	0.89

Note. Interaction Coef/OR is the associated regression models regression coefficient (or exponentiate of the coefficient) for the interaction term. PP refers to per-protocol and ITT to intention-to-treat. MET = MET minutes; V/f = vegetable/fruit; St = Sought treatment. MET minutes are rounded to zero decimal places, and all other variables to 2 decimal places.

Figure 2. Estimated conditional means and 95 % CI for the imputed PP models with treatment/control interaction by risk level

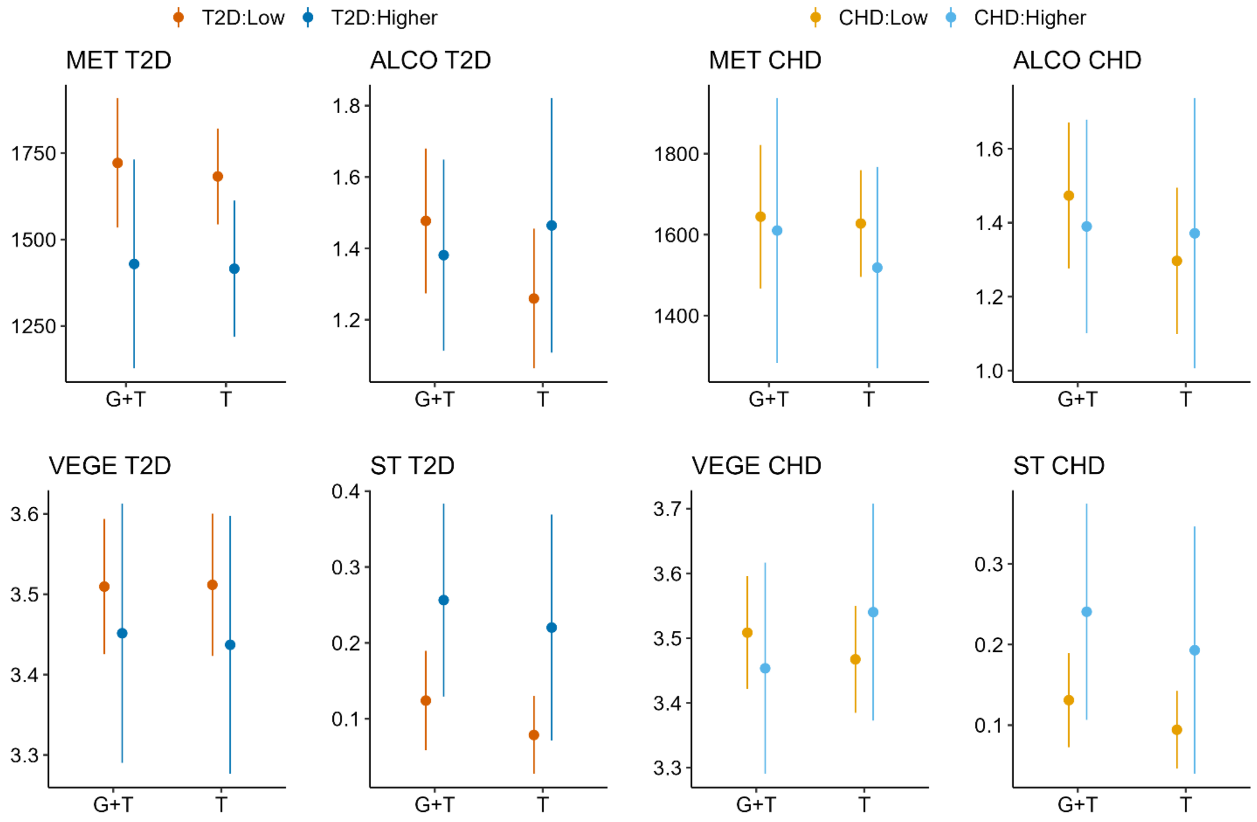

Note. “MET” = average MET minutes (per w.), “ALCO” = average alcohol portions (per w.), “VEGE” = composite score for vegetable and fruit consumption, “ST” = did the participant seek medical treatment/examination after seeing the P5 results (probability), “Low risk” = less than 7.5 % risk of developing T2D/CHD during the next ten years, and “High risk” = more than 7.5 % risk of developing T2D/CHD during the next ten years. G+T = Genetic and traditional (experimental group), and T = Traditional (control group). PP = per-protocol.

Figure 3. Estimated conditional means and 95 % CI for the imputed ITT models with treatment/control interaction by risk level

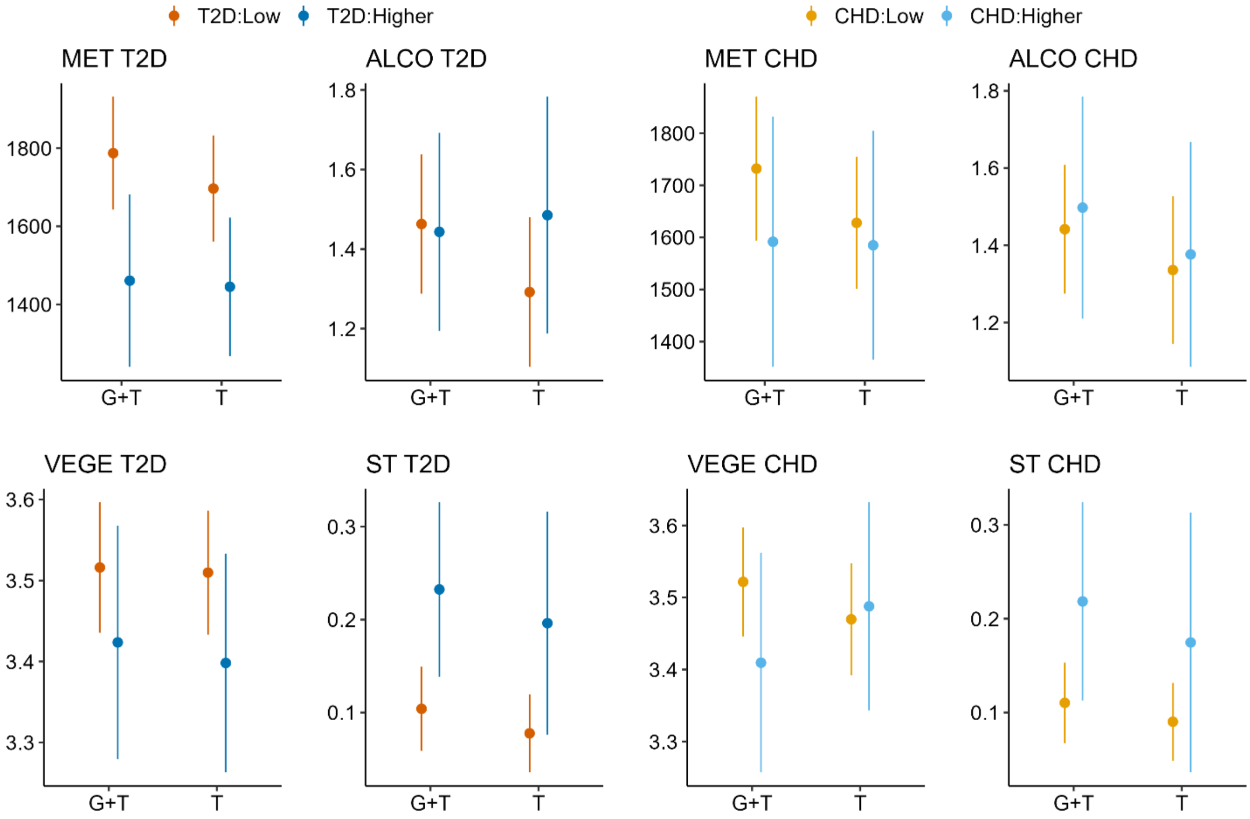

Note. "MET" = average MET minutes (per w.), "ALCO" = average alcohol portions (per w.), "VEGE" = composite score for vegetable and fruit consumption, "ST" = did the participant seek medical treatment/examination after seeing the P5 results (probability), "Low risk" = less than 7.5 % risk of developing T2D/CHD during the next ten years, and "High risk" = more than 7.5 % risk of developing T2D/CHD during the next ten years. G+T = Genetic and traditional (experimental group), and T = Traditional (control group). ITT = intention-to-treat.

The imputed results resembled, for the most part, those obtained with complete case analysis. PP with complete cases had very similar mean differences and estimated means for the MET minutes, alcohol consumption, and vegetable and fruit consumption compared to PP analysis with imputed data. Results concerning whether the respondents sought treatment varied slightly. The imputed analysis had a higher estimated mean for both the treatment and the control group. Likewise, the mean difference was somewhat higher between the groups (although not enough to change the conclusions drawn). A similar pattern was observed when comparing the imputed ITT and complete case ITT analyses. MET minutes, alcohol consumption, and vegetable and fruit consumption showed very similar estimated means and mean differences between the analyses, while the sought treatment variable displayed a similar trend that was observed with PP analyses. The difference between CC and imputed analysis in relation to the sought treatment variable reflects combination

of the auxiliary variables' influence on the imputation model since almost all of the auxiliary variables had some association with missingness. However, the slight differences do not change any of the conclusions made based on the CC analysis. The interaction models also produced relatively similar results when the imputed and CC analyses were compared with no noteworthy differences.

References

Buuren S van (2021) Flexible imputation of missing data, Second edition. Chapman & Hall/CRC, Boca Raton

Buuren S van, Groothuis-Oudshoorn K (2011) mice: Multivariate Imputation by Chained Equations in R. Journal of Statistical Software 45:1–67. https://doi.org/10.18637/jss.v045.i03

Croux C, Dhaene G, Hoorelbeke D. (2003) Robust standard errors for robust estimators. Discussion Papers Series 03.16, K.U. Leuven, CES.

Li P, Stuart EA (2019) Best (but oft-forgotten) practices: missing data methods in randomized controlled nutrition trials. Am J Clin Nutr 109:504–508. https://doi.org/10.1093/ajcn/nqy271

Sullivan TR, White IR, Salter AB, Ryan P, Lee KJ (2018) Should multiple imputation be the method of choice for handling missing data in randomized trials? Stat Methods Med Res 27:2610–2626. https://doi.org/10.1177/0962280216683570
